# Characteristics and prognosis of patients with COVID-19 and hematological diseases in Japan: a cross-sectional study

**DOI:** 10.1007/s12185-023-03685-w

**Published:** 2024-01-03

**Authors:** Daisuke Minakata, Tomoyuki Uchida, Aya Nakano, Ken Takase, Nodoka Tsukada, Hiroshi Kosugi, Eri Kawata, Takahiko Nakane, Hiroyuki Takahashi, Tomoyuki Endo, Satoshi Nishiwaki, Hideaki Fujiwara, Akiko M. Saito, Toshiki I. Saito, Koichi Akashi, Itaru Matsumura, Kinuko Mitani

**Affiliations:** 1 Division of Hematology, Department of Medicine, Jichi Medical University, Tochigi, Japan; 2 Department of Hematology, Eiju General Hospital, Tokyo, Japan; 3 Division of Clinical Oncology and Hematology, Department of Internal Medicine, The Jikei University School of Medicine Tokyo, Tokyo, Japan; 4 Department of Haematology, Clinical Research Centre, National Hospital Organization Kyushu Medical Centre, Fukuoka, Japan; 5 Department of Hematology/Oncology, Asahikawa Kosei General Hospital, Asahikawa, Japan; 6 Department of Hematology, Ogaki Municipal Hospital, Ogaki, Japan; 7 Department of Hematology, Panasonic Health Insurance Organization Matsushita Memorial Hospital, Moriguchi, Japan; 8 Department of Hematology, Osaka Saiseikai Nakatsu Hospital, Osaka, Japan; 9 Department of Hematology and Medical Oncology, Kanagawa Cancer Center, Kanagawa, Japan; 10 Department of Hematology, Faculty of Medicine, Hokkaido University, Sapporo, Japan; 11 Department of Hematology and Oncology, Nagoya University Graduate School of Medicine, Nagoya, Japan; 12 Department of Hematology and Oncology, Okayama University Hospital, Okayama, Japan; 13 Clinical Research Center, National Hospital Organization Nagoya Medical Center, Nagoya, Japan; 14 Department of Medicine and Biosystemic Science, Kyushu University Graduate School of Medical Science, Fukuoka, Japan; 15 Department of Hematology and Rheumatology, Faculty of Medicine, Kindai University, Osakasayama, Japan; 16 Department of Hematology and Oncology, Dokkyo Medical University, 880, Kitakobayashi, Mibu-machi, Shimotsuga-gun, Tochigi, 321-0293 Japan

**Keywords:** COVID-19, Hematological disease, Japan, Japanese Society of Hematology

## Abstract

The Japanese Society of Hematology performed an observational cross-sectional study to clarify the morbidity, prognosis, and prognostic factors in patients with COVID-19 with hematological diseases (HDs) in Japan. The study included patients with HDs who enrolled in our epidemiological survey and had a COVID-19 diagnosis and a verified outcome of up to 2 months. The primary endpoints were characteristics and short-term prognosis of COVID-19 in patients with HDs. A total of 367 patients from 68 institutes were enrolled over 1 year, and the collected data were analyzed. The median follow-up among survivors was 73 days (range, 1–639 days). The 60-day overall survival (OS) rate was 86.6%. In the multivariate analysis, albumin ≤ 3.3 g/dL and a need for oxygen were independently associated with inferior 60-day OS rates (hazard ratio [HR] 4.026, 95% confidence interval (CI) 1.954–8.294 and HR 14.55, 95% CI 3.378–62.64, respectively), whereas 60-day survival was significantly greater in patients with benign rather than malignant disease (HR 0.095, 95% CI 0.012–0.750). Together, these data suggest that intensive treatment may be necessary for patients with COVID-19 with malignant HDs who have low albumin levels and require oxygen at the time of diagnosis.

**Supplementary Information:**

The online version contains supplementary material available at 10.1007/s12185-023-03685-w.

## Introduction

According to a World Health Organization report, the coronavirus disease (COVID-19) pandemic started at the end of 2019, and 768,560,727 cases and 6,952,522 deaths were confirmed globally as of July 31, 2023 [1]. According to COVID-19 REGISTRY JAPAN [COVIREGI-JP] data [2] for all hospitalized cases of COVID-19 in Japan, 61.6% of the 2638 patients received no oxygen, whereas 29.9% required oxygen administration, and 8.5% of patients required invasive mechanical ventilation or management by extracorporeal membrane oxygenation (ECMO). A total of 7.5% (197/2634) of all patients died within approximately 4 months after the data entry period starting in March 2020. Reports from other countries such as China [3], the USA [4], and European countries [5–9] have stated that patients with hematological diseases (HDs) have poor prognoses, and the fatality rate in these patients tends to be higher than that in patients without HDs. However, the influences of various HDs and related therapies on the prognosis of patients with COVID-19 remain largely unknown. Notably, treatments for HDs, such as cytotoxic agents, immunomodulators, hematopoietic stem cell transplantation, and chimeric antigen receptor T-cell therapy, are highly immunosuppressive. Furthermore, many patients with hematological malignancies have additional risk factors of particular concern in the context of COVID-19, including advanced age, as well as underlying or treatment-induced comorbid illnesses, such as hypertension, diabetes, and chronic lymphopenia. Interestingly, complications of COVID-19 include hypercoagulability and thrombosis, which can have substantial consequences for people with cancer who are already at high risk of venous thromboembolic events [10, 11]. The American Society of Hematology (ASH) has provided an online registry of patients with COVID-19 with in-depth inquiries in the ASH Research Collaborative Data Hub [4]. The reported data for the 250 initially enrolled patients have indicated that the severity of COVID-19 in patients with HDs was mild in 31% (n = 77), moderate in 38% (n = 96), and severe in 29% (n = 72), and the overall mortality rate was 28%. In Japan, Uchida et al. [12], at Eiju General Hospital, Tokyo, have retrospectively analyzed data for patients with HDs who developed COVID-19 as a nosocomial infection. In that study, 21 of 40 patients died, and this mortality rate was higher than that among patients with other diseases who were hospitalized in other departments in the same hospital (20/57 died). However, no large-scale studies have reported SARS-CoV-2 infections in patients with benign or malignant HDs in Japan. The Japanese Society of Hematology (JSH) has noted that collecting and evaluating these data would aid in the selection of appropriate treatments for patients with HDs in the COVID-19 pandemic. Therefore, this study was aimed at investigating the characteristics and prognosis of patients with COVID-19 with HDs in Japan.

## Materials and methods

### Study design and participants

This multicenter, observational, cross-sectional study was conducted at 68 institutes in Japan. The COVID-19 registry research group worked on behalf of the JSH. The JSH-COVID-19–20 Registry collected data between June 2021 and May 2022. The data were fixed for analyses on August 15, 2022. The scheduled registration and analysis periods were 1 year each, for a total of 2 years, following the Ethical Review Committee’s clearance. The inclusion criteria were as follows: (1) patients who had participated, or would participate, in the JSH epidemiological survey “Hematological Disease Registration” [13]; and (2) patients diagnosed with COVID-19 (SARS-CoV-2 polymerase chain reaction (PCR)-positive or antigen-positive), with a confirmed prognosis up to 2 months after diagnosis. Patients with a suspected diagnosis based on imaging or antibody testing were not included. Patients were also excluded if the lead investigator or sub-investigators deemed them unsuitable, or if the patient or the patient’s guardian declined to participate. After completing the study, we conducted an additional survey at the participating sites between June 5, 2023, and June 19, 2023, to clarify the capture rate among patients. Each participating site was asked to provide the number of unenrolled patients with definitive diagnosis of COVID-19 and a known 2-month prognosis at the end of registration (May 31, 2022) and the associated HDs. The registry and analysis were reviewed and approved by the Public Health Research Foundation Institutional Review Board (IRB). After IRB approval was obtained from the principal investigator’s facility (Dokkyo Medical University), the data providers (institutions) were required to obtain approval from their respective IRBs. All procedures complied with the General Data Protection Regulation requirements. The study protocols were performed according to the principles of the Declaration of Helsinki. This investigation was registered in Japan as a clinical trial (UMIN000044254) [14].

### Procedures and outcomes

The primary endpoint of this analysis was overall survival (OS) at 60 days after a COVID-19 diagnosis. COVID-19 was diagnosed by the institution, and the diagnostic method (PCR test and/or antigen test) and SARS-CoV-2 infection route were recorded. The secondary objectives included the underlying disease and treatment status before SARS-CoV-2 infection, details of SARS-CoV-2 infection, treatment response. The JSH-COVID-19–20 Registry collected information on the patients’ baseline characteristics and clinical findings, treatments, and outcomes of COVID-19 relevant to HDs. The HDs investigated in this study were those included in the JSH epidemiological survey [13] “Registration of Hematological Diseases.”

### Periods and strains of COVID-19 in each wave

Major strains of COVID-19 observed and defined period for each wave in this study are as follows. First wave (January 29, 2020, to June 13, 2020), European strain (B.1.1): second wave (June 14, 2020, to October 9, 2020), variant of European strain (B.1.1.284); third wave (October 10, 2020, to February 28, 2021), variant of European strain (B.1.1.214);fourth wave (March 1, 2021, to June 20, 2021), Alpha strain (B1.1.7); fifth wave (June 21, 2021, to December 16, 2021), Delta strain (AY.29); sixth wave (December 17, 2021, to June 24, 2022), Omicron strain (BA.1.1.2) [15].

### Statistical analysis

Patient features were compared with Fisher’s exact test for categorical variables and the Mann–Whitney U test or *t*-test for continuous variables. OS was defined as the time from COVID-19 diagnosis to death or the date of the last follow-up (as many as 60 days). Probabilities of OS were calculated according to the Kaplan–Meier method and compared with the log-rank test. The predictive abilities and ideal cutoff values of laboratory data for OS were evaluated through receiver-operating characteristic (ROC) curve and area under the ROC curve (AUC) analyses. The influence of these parameters on OS was then assessed with the Cox proportional hazard model. Variables with P < 0.20 in univariate analysis were included as independent variables in multivariate analysis. All statistical analyses were performed in EZR (Saitama Medical Center, Jichi Medical University, Saitama, Japan), a customized version of R Commander designed to include statistical functions often used in biostatistics [16].

## Results

### Patient background and comorbidities

Data for 367 patients from 68 facilities were included in our analysis. Our subsequent survey revealed that 150 unenrolled patients had a confirmed prognosis of 2 months after the diagnosis of COVID-19. Among these patients, the number of reported HDs was 25. The capture rate for COVID-19 cases within the registration period was 71.0%.

The baseline patient characteristics and comorbidities are illustrated in Table 1. The median age at the time of COVID-19 diagnosis was 69 years (range 18–95 years); 215 patients (58.6%) were male, and 152 patients (41.4%) were female, one of whom was pregnant. Current or previous smokers comprised 34.3% of the patients, but no patients reported any experience with vaping (or no information was available). The number of patients who had been vaccinated against COVID-19 was 110 (30.0%). The diagnosis of COVID-19 was verified by PCR-based testing in 325 (88.6%) reported cases and by antigen testing in 54 (14.7%) reported cases. Most cases of SARS-CoV-2 infection were community-acquired (n = 227; 61.9%) or nosocomial (n = 88; 23.9%); the infection route in the remaining patients was unclear. A total of 106 patients (28.9%) reported more than one comorbidity, among which hypertension was most frequent (28.9%) and was followed by diabetes (19.4%). Twenty-four patients (6.5%) participated in other registries, all of whom were enrolled in COVIREGI-JP. Details of laboratory and imaging tests at the time of COVID-19 diagnosis are described in Supplemental Table S1. The most prevalent finding on chest X-ray or CT images of the lungs was ground-glass opacity or ground-glass attenuation (81.9%), and unilateral and bilateral findings were observed in 14.5% and 85.5% of cases, respectively.

**Table 1:** Patient characteristics and comorbidities

Patient background	Total patients (n = 367)
Age, median (range), years	69 (18–95)
Performance status	
0/1/2/3/4	160 (43.6%)/119 (32.4%)/45 (12.3%)/32 (8.7%)/11 (3.0%)
Male/female, n (%)	215 (58.6%)/152 (41.4%)
Pregnancy at diagnosis	1/152 (0.7%)
Height/weight, median (range), cm/kg (n = 364)	161.2 (135–188)/58.5 (25.7–116.4)
BMI, median (range), kg/m^2^	22.2 (13.7–39.4)
Smoking status	
Current/former/never/unknown, n (%)	15 (4.1%)/111 (30.2%)/171 (46.6%)/70 (19.1%)
Vaping status	
Current/former/never/unknown, n (%)	0 (0.0%)/0 (0.0%)/227 (61.8%)/140 (38.2%)
Vaccinated patients with COVID-19, n (%)	110 (30.0%)
COVID-19 diagnosis confirmation methods	
Real time PCR test/other PCR methods, n (%)	303 (82.6%)/22 (6.0%)
Antigen test/quantity test, n (%)	54 (14.7%)/43 (11.7%)
Presumed route of SARS-CoV-2 infection	
Community/nosocomial infection, n (%)	227 (61.9%)/88 (23.9%)
Others/unknown, n (%)	25 (6.8%)/27 (7.4%)
Patient comorbidities	
Rheumatoid arthritis, n (%)	24 (6.5%)
Chronic kidney disease, n (%)	40 (10.9%)
Congestive heart failure, n (%)	18 (4.9%)
COPD/emphysema, n (%)	23 (6.3%)
Coronary artery disease, n (%)	15 (4.1%)
Diabetes, n (%)	71 (19.4%)
Hepatitis B or C, n (%)	26 (7.1%)
Hepatic dysfunction, n (%)	28 (7.6%)
Hypertension, n (%)	106 (28.9%)
Cerebral infarction/transient ischemic attack, n (%)	17 (4.6%)
Non-hematologic cancer/lung cancer, n (%)	31 (8.5%)/6 (1.6%)
Primary/secondary hypogammaglobulinemia, n (%)	69 (18.8%)
IgG at diagnosis, median (range), mg/dl (n = 186)	923 (132–4398)
HIV infection, n (%)	3 (0.8%)
Venous thromboembolism (VTE)	6 (1.6%)
Time since VTE, median (range), days (n = 6)	77 (32–667)
Factors triggering the latest VTE (n = 6)	
Yes/none/unknown, n	4/1/1
Treatment at diagnosis (n = 6)	
Observation/edoxaban	2/4

*BMI* body mass index, *PCR* polymerase chain reaction, *COPD* chronic obstructive pulmonary disease, *HIV* human immunodeficiency virus

### Background HDs and treatment

The HDs at the time of COVID-19 diagnosis included seven benign (n = 43; 11.7%) and 25 malignant diseases (n = 324; 88.3%; Table 2). The median time between HD and COVID-19 diagnoses was 710 days (benign disease 1,046 days; malignant disease 670 days). The most common benign HD was immune thrombocytopenia (ITP), which accounted for 5.5% (n = 20) of the total population. The most common malignant HD was aggressive B-cell lymphoma, which was followed by multiple myeloma and myelodysplastic syndromes. Malignant lymphoma, including aggressive B-cell lymphoma, affected 145 people, accounting for approximately 40% of the entire population. The composition of each HD and the details of the treatments for each disease in the last year before the COVID-19 diagnosis and at the time of the COVID-19 diagnosis are shown in Supplemental Fig. S1 and Table S2 (1)–(33). Allogeneic and autologous transplantation were conducted in 36 (9.8%) and 16 (4.4%) patients, respectively, and none of the patients were treated with CAR-T (CD19 CAR-T). Table 3 provides information on HD status at the time of COVID-19 diagnosis, and the time between final treatment and diagnosis. At the time of COVID-19 diagnosis, 14.7% of patients were in initial diagnosis/on induction therapy; 18.8% were in remission on consolidation or maintenance therapy; 24.8% were in remission and not on therapy; 20.4% had stable disease but were not in remission; and 16.4% had a status of relapsed and refractory. Regarding the time from the last treatment for HDs to COVID-19 diagnosis, most patients (n = 211; 57.5%) were currently undergoing treatment; times within 3 months, within 3–6 months, within 6 months to 1 year, and within 1–2 years accounted for less than 10% each, and times after 2 years accounted for 14.7%.

**Table 2:** Background hematological diseases and history of cellular therapies

Variable	Total patients (n = 367)
Time from HD diagnosis to COVID-19 diagnosis, median (range), days	710 (– 121 to 9778)
Benign disease^a^	43 (11.7%)
Aplastic anemia, n (%)	9 (2.5%)
Warm AIHA/CAD, n (%)	4 (1.1%)/1 (0.3%)
PNH, n (%)	3 (0.8%)
ITP, n (%)	20 (5.5%)
PRCA, n (%)	4 (1.1%)
Thalassemia, n (%)	1 (0.3%)
TTP, n (%)	1 (0.3%)
Malignant disease^a^	324 (88.3%)
MDS, n (%)	36 (9.8%)
MPN (PV, ET)/CMML, n (%)	12 (3.3%)/3 (0.8%)
MPN (MF), n (%)	4 (1.1%)
CML, n (%)	17 (4.6%)
AML/APL/AUL, n (%)	25 (6.8%)/8 (2.2%)/1 (0.3%)
Ph negative B-ALL/Ph positive B-ALL, n (%)/MPAL, n (%)	5 (1.4%)/6 (1.6%)/1(0.3%)
T-ALL, n (%)	1 (0.3%)
Aggressive B-cell lymphoma/indolent BL/mantle cell lymphoma, n (%)	70 (19.1%)/35 (9.5%)/5 (1.4%)
MM/LPL, n (%)	46 (12.5%)/7 (1.9%)
PCNSL, n (%)	7 (1.9%)
CLL, n (%)	3 (0.8%)
POEMS, n (%)	3 (0.8%)
Aggressive T-cell lymphoma/indolent TL/ATLL, n (%)	9 (2.5%)/4 (1.1%)/3 (0.8%)
HL, n (%)	12 (3.3%)
ENTL, n (%)	1 (0.3%)
Cellular therapies (transplantation and chimeric antigen receptor T-cell therapy)	
Time from transplantation to COVID-19 diagnosis, median (range), days	776 (-201 to 7368)
Allogeneic SCT, n (%)	36 (9.8%)
Transplantation source BM/PB/CBT, n (%)	17 (47.2%)/13 (36.1%)/6 (16.7%)
Matched related/matched unrelated/mismatched unrelated/haploidentical SCT	11(30.6%)/11(30.6%)/9(25.0%)/5 (13.8%)
GVHD acute/chronic, n (%)	4 (11.1%)/13 (36.1%)
GVHD therapy, n (%) n = 27	16 (59.3%)
Autologous SCT, n (%)	16 (4.4%)
Chimeric antigen receptor, n (%)	0 (0.0%)

*HD* hematological disease, *AIHA* autoimmune hemolytic anemia, *CAD* cold agglutinin disease, *PNH* paroxysmal nocturnal hemoglobinuria, *ITP* idiopathic thrombocytopenic purpura, *PRCA* pure red cell aplasia, *TTP* thrombotic thrombocytopenic purpura, *MDS* myelodysplastic syndrome, *MPN* myeloproliferative neoplasm, *CMML* chronic myelomonocytic leukemia, *MF* myelofibrosis, *CML* chronic myeloid leukemia, *AML* acute myeloid leukemia, *APL* acute promyelocytic leukemia, *AUL* acute undifferentiated leukemia, *Ph* Philadelphia, *ALL* acute lymphocytic leukemia, *MPAL* mixed-phenotype acute leukemia, *ATLL* adult T-cell leukemia-lymphoma, *HL* Hodgkin lymphoma, *PCNSL* primary central nervous system lymphoma, *MM* multiple myeloma, *LPL* lymphoplasmacytic lymphoma, *CLL* chronic lymphocytic leukemia, *ENTL* extranodal NK/T cell lymphoma, *SCT* stem cell transplantation, *BM* bone marrow, *PB* peripheral blood, *CBT* cord blood transplantation, *GVHD* graft-versus-host disease

^a^The HDs investigated in this study were those included in the JSH’s epidemiological survey [13] “Registration of Hematological Diseases.”

**Table 3:** Hematologic disease status at the time of diagnosis of COVID-19 and time since treatment for hematologic disease

Variable	Total patients (n = 367)
Disease status at COVID-19 diagnosis	
Initial diagnosis/on induction treatment, n (%)	54 (14.7%)
In remission on consolidation or maintenance treatment, n (%)	69 (18.8%)
In remission not on treatment, n (%)	91 (24.8%)
Stable but not in remission, n (%)	75 (20.4%)
Relapsed or refractory, n (%)	60 (16.4%)
Other/unknown, n (%)	17 (4.6%)/1 (0.3%)
Time from the last therapy for HD to COVID-19 diagnosis	
Currently undergoing treatment, n (%)	211 (57.5%)
Within the past 3 months before COVID-19 diagnosis, n (%)	34 (9.3%)
3–6 months before COVID-19 diagnosis, n (%)	7 (1.9%)
6 months to 1 year before COVID-19 diagnosis, n (%)	22 (6.0%)
1–2 years before COVID-19 diagnosis, n (%)	19 (5.2%)
> 2 years before COVID-19 diagnosis, n (%)	54 (14.7%)
Unknown, n (%)	20 (5.5%)

### Presenting symptoms and severity at the time of COVID-19 diagnosis

We examined the presenting symptoms of patients with COVID-19 (Table 4). The most prevalent presenting symptoms were fever (85.0%), cough (50.6%), shortness of breath (31.0%), and fatigue (29.4%). We also examined the severity COVID-19 diagnosis according to the ASH [4] and Ministry of Health, Labour and Welfare (MHLW) [15] criteria. According to the ASH criteria, 127 (34.6%), 220 (60.0%), and 20 (5.4%) patients had mild, moderate, and severe disease, respectively. According to the MHLW criteria, 182 (49.6%), 87 (23.7%), 80 (21.8%), and 18 (4.9%) were classified as having mild, moderate-I, moderate-II, and severe disease, respectively.

**Table 4:** Symptoms and severity at the time of COVID-19 diagnosis

Symptoms of COVID-19 (n = 326)	
Fever ≥ 37.5, n (%)	277 (85.0%)
Shortness of breath, n (%)	101 (31.0%)
Cough, n (%)	165 (50.6%)
Diaphoresis, n (%)	33 (10.1%)
Anosmia, n (%)	24 (7.4%)
Rhinorrhea, n (%)	32 (9.8%)
Dysgeusia, n (%)	45 (13.8%)
Abdominal pain, n (%)	5 (1.5%)
Diarrhea, n (%)	16 (4.9%)
Nausea, vomiting, n (%)	9 (2.8%)
Confusion, n (%)	5 (1.4%)
Fatigue, n (%)	96 (29.4%)
Headache, n (%)	39 (12.0%)
Myalgia, n (%)	14 (4.3%)
Weight loss, n (%)	7 (2.1%)
Other, n (%)	58 (17.8%)
Saturation and severity (ASH/MHLW)	
Oxygen saturation, median (range), % (n = 313)	96 (65–100)
Severity of COVID-19 (ASH)*	
Mild, n (%)	127 (34.6%)
Moderate, n (%)	220 (60.0%)
Severe, n (%)	20 (5.4%)
Severity of COVID-19 (MHLW)^a^	
Mild, n (%)	182 (49.6%)
Moderate-I, n (%)	87 (23.7%)
Moderate-II, n (%)	80 (21.8%)
Severe, n (%)	18 (4.9%)

*ASH* American Society of Hematology, *MHLW* Ministry of Health, Labour and Welfare in Japan

^a^The severity was classified by the ASH [4] as mild (outpatient level), moderate (hospitalization level), or severe (intensive care unit [ICU] level), and by the MHLW [15] in Japan as mild (SPO_2_ ≥ 96% and/or no respiratory symptoms), moderate-I (93% < SPO_2_ < 96% and/or dyspnea, pneumonia findings), moderate-II (SPO_2_ < 93% and/or situations needing oxygen administration), or severe (ICU admission and/or ventilator management)

### Treatment and supportive care for COVID-19

COVID-19-specific therapies for all patients are shown in Supplemental Table S3. The most common COVID-19-specific medications were remdesivir (40.3%) and dexamethasone including alternate glucocorticoids (37.3%), followed by favipiravir (15.5%) and tocilizumab (6.3%). However, approximately 20% of all patients received no treatment or observation.

We also explored the use of supportive care for COVID-19. Approximately 60% of patients did not receive oxygen at the time of COVID-19 diagnosis, and approximately 40% of patients required low-flow oxygen (1–5 L). Less than 10% of patients required invasive mechanical ventilation, and only 3% required ECMO. Approximately one-quarter (24.3%) of patients were given anticoagulant medication at the time of COVID-19 diagnosis, most commonly unfractionated heparin (n = 53; 14.4%).

### Laboratory data predicting OS

**Fig. 1:**
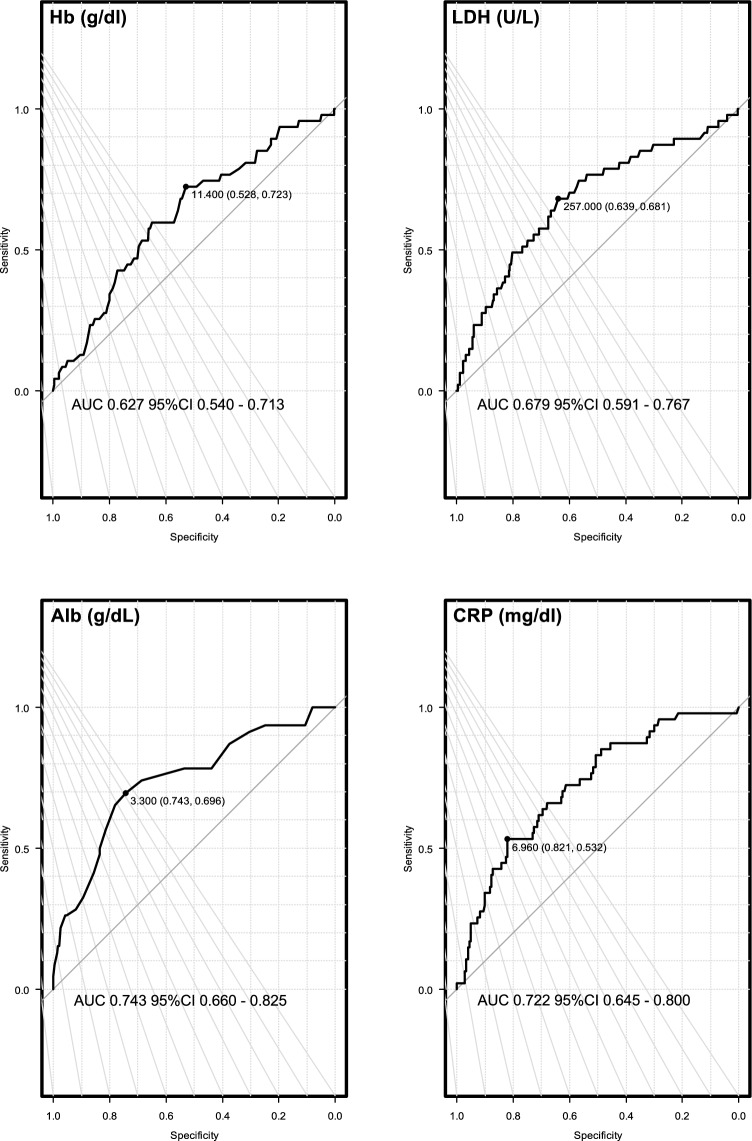
Receiver operating characteristic curve analysis of laboratory data at the time of COVID-19 diagnosis for overall survival in all patients. *ROC* receiver operating characteristic, *AUC* area under the curve, *Hb* hemoglobin, *LDH* lactate dehydrogenase, *Alb* albumin, *CRP* C-reactive protein

Boxplots of the laboratory test results at the time of COVID-19 diagnosis are shown in Supplemental Fig. S2, stratified by survival status in all patients. Laboratory indicators at the time of COVID-19 diagnosis, including hemoglobin, albumin, lactate dehydrogenase, and C-reactive protein (CRP), showed substantial differences between the patients who survived and those who died. We then used ROC curve analysis to assess the abilities of these four factors to predict OS outcomes (Fig. 1). Albumin and CRP had a relatively high predictive value compared to other factors for all patients (threshold 3.3, AUC 0.743, 95% CI 0.660–0.825; threshold 6.96, AUC 0.722, 95% CI 0.645–0.800).

### Outcomes of SARS-CoV-2 infection

**Fig. 2:**
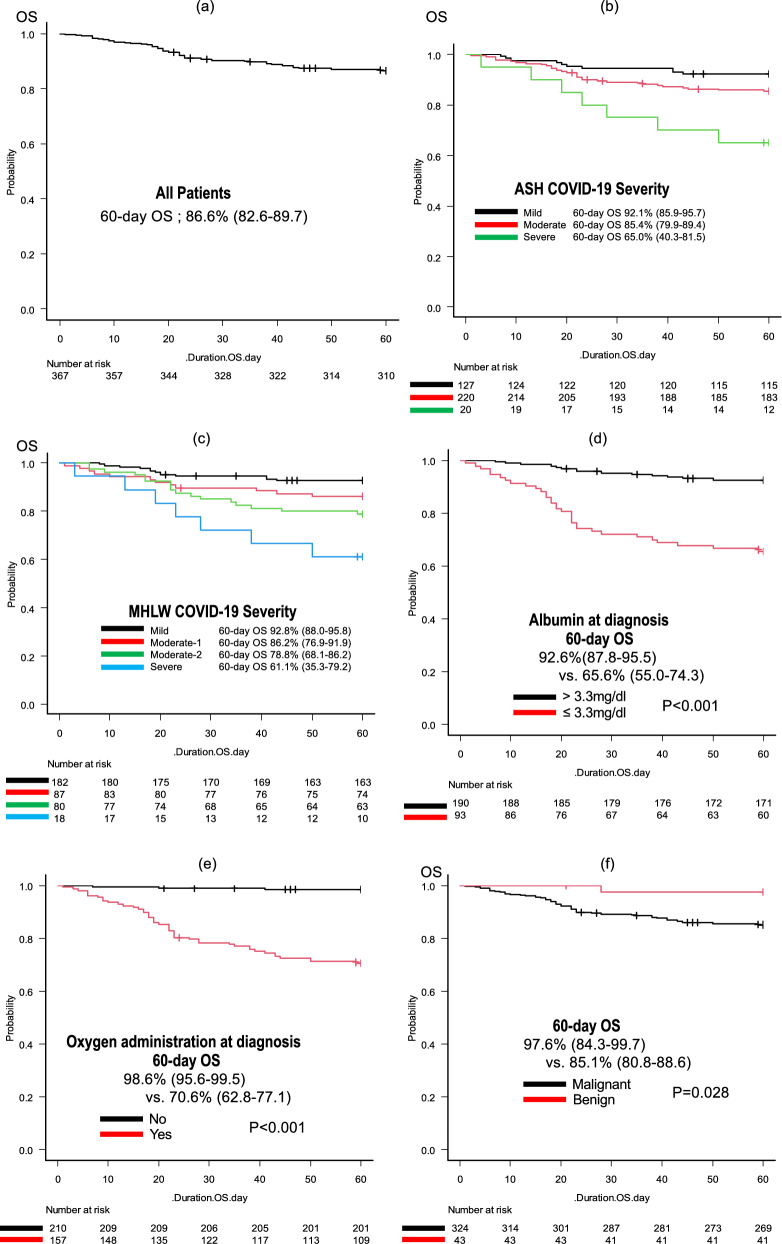

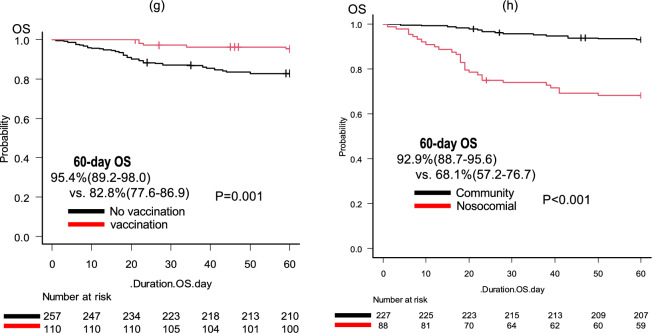
Kaplan–Meier estimates of overall survival. Kaplan–Meier estimates of overall survival for **a** all patients and with stratification by **b** ASH severity, **c** JMHW severity, **d** albumin at diagnosis, **e** oxygen required, **f** benign or malignant hematological diseases, **g** vaccination status, and **h** route of infection. *ASH* The American Society of Hematology, *MHLW* The Ministry of Health, Labour and Welfare in Japan

The median follow-up duration for the survivors was 73 days (range 1–639). At the data cutoff, 49 (13.3%) patients had died. Among the entire patient population, the 60-day OS rate was 86.6% (95% CI 82.6–89.7%), and the median OS was not reached (Fig. 2a). In the ASH classification, the 60-day OS rates were 92.1% (95% CI 85.9–95.7%), 85.4% (95% CI 79.9–89.4%), and 65.0% (95% CI 40.3–81.5%) for the mild, moderate, and severe groups, respectively (Fig. 2b). For the MHLW classification, the 60-day OS rates were 92.8% (95% CI 88.0–95.8%), 86.2% (95% CI 76.9–91.9%), 78.8% (95% CI 68.1–86.2%), and 61.1% (95% CI 35.3–79.2%) for the mild, moderate-I, moderate-II, and severe groups, respectively (Fig. 2c). Furthermore, univariate analysis indicated that several factors were associated with OS, including age > 60, Alb ≤ 3.3 g/dl, severity according to ASH classification, severity according to MHLW classification, benign disease, PS > 2, relapsed/refractory status, oxygen requirement at diagnosis, and CRP > 7.0 mg/dl (P = 0.006, P < 0.001, P = 0.003, P < 0.001, P = 0.028, P < 0.001, P < 0.001, P < 0.001, and P < 0.001, respectively; Table 5). In the multivariate analysis considering these variables, Alb ≤ 3.3 g/dl (hazard ratio (HR) 4.026, 95% CI 1.954–8.294, P < 0.001) and requiring oxygen (HR 14.55, 95% CI 3.378–62.64, P < 0.001) were independently associated with shorter OS, whereas benign disease (HR 0.095, 95% CI 0.012–0.750,* P* = 0.026) was associated with longer OS. The multivariate analysis in the patients with malignant HDs, excluding benign HDs, also indicated that Alb ≤ 3.3 g/dl (hazard ratio (HR) 4.165, 95% CI 2.002–8.666, P < 0.001) and requiring oxygen (HR 14.72, 95% CI 3.421–63.33, P < 0.001) were independently associated with shorter OS (Supplemental Table S4). The presence of HDs, such as lymphoma, was not in itself a significant factor associated with OS in either cohort.

**Table 5:** Univariate and multivariate analyses of 60-day OS

Risk factor	Univariate analysis		Multivariate analysis	
	60-day OS [%], 95%CI	P-value	HR (95%CI)	P-value
Age (> 60)	83.1 (77.8–87.3)	0.006		0.726
(≤ 60)	93.5 (87.3–96.7)		Reference	
Alb (≤ 3.3 g/dl)	65.6 (55.0–74.3)	< 0.001	4.026 (1.954–8.294)	< 0.001
(> 3.3 g/dl)	92.6 (87.8–95.5)		Reference	
ASH classification (severe)	65.0 (40.3–81.5)	0.003		0.996
(mild and moderate)	87.8 (83.9–90.9)		Reference	
MHLW classification (severe)	61.1 (35.3–79.2)	< 0.001		0.996
(mild and moderate I, II)	87.9 (84.0–90.9)		Reference	
BMI (> 30 kg/m^2^)	77.8 (36.5–93.9)	0.487		
(≤ 30 kg/m^2^)	86.7 (82.7–89.8)			
Current or former smoking	88.0 (80.8–92.6)	0.562		
Never	85.9 (80.8–89.7)			
Benign disease	97.6 (84.3–99.7)	0.028	0.095 (0.012–0.750)	0.026
Malignant	85.1 (80.8–88.6)		Reference	
MDS	80.6 (63.5–90.2)	0.254		
No MDS	87.2 (83.1–90.4)			
Leukemia	89.1 (75.8–95.3)	0.570		
No leukemia	86.2 (81.9–89.6)			
Lymphoma	83.5 (76.0–88.8)	0.190		0.924
No lymphoma	88.4 (83.5–91.9)		Reference	
PCD	81.4 (68.9–89.2)	0.196		0.331
No PCD	87.6 (83.4–90.8)		Reference	
PS (> 2)	69.1 (58.2–77.6)	< 0.001		0.340
(≤ 2)	92.1 (88.2–94.7)		Reference	
Relapsed or refractory	68.3 (55.0–78.5)	< 0.001		0.128
Except for the above	90.2 (86.3–93.0)		Reference	
Ongoing therapy at diagnosis	85.2 (79.7–89.4)	0.379		
Except for the above	88.4 (82.2–92.5)			
Oxygen required	70.6 (62.8–77.1)	< 0.001	14.55 (3.378–62.64)	< 0.001
Except for the above	98.6 (95.6–99.5)		Reference	
Neutrophils (< 1000/µL)	92.1 (77.5–97.4)	0.155		0.082
(≥ 1000/µL)	83.1 (77.9–87.2)		Reference	
Lymphocytes (< 1000/µL)	82.2 (76.2–86.8)	0.158		0.638
(≥ 1000/µL)	89.1 (80.6–94.0)		Reference	
CRP (> 7.0 mg/dl)	64.5 (51.9–74.6)	< 0.001		0.163
(≤ 7.0 mg/dl)	89.7 (85.0–93.1)		Reference	

*OS* overall survival, *ASH* American Society of Hematology, *MHLW* Ministry of Health Labour and Welfare in Japan, *BMI* body mass Index, *MDS* myelodysplastic syndromes, *PCD* plasma cell dyscrasia, *PS* performance status, *CRP* C-reactive protein

The 60-day OS rates for patients with Alb ≤ 3.3 g/dl and oxygen required (65.6%; 95% CI 55.0–74.3% and 70.6%; 95% CI 62.8–77.1%) were inferior to those of other patients (92.6%; 95% CI 87.8–95.5%; P < 0.001 and 98.6%; 95% CI 95.6–99.5%; P < 0.001) (Fig. 2d, e). In contrast, patients with benign disease had a longer OS than patients with malignant disease (60-day OS; 97.6%; 95% CI 84.3–99.7% vs. 85.1%; 95% CI 80.8–88.6%, P = 0.028; Fig. 2f). We also compared OS between vaccinated and non-vaccinated patients, as well as between patients with community-acquired and nosocomial infections. As expected, the OS was longer in vaccinated patients and those with community-acquired infection than in the other patients (60-day OS; 95.4% vs. 82.8%, P = 0.001 and 92.9% vs. 68.1%, P < 0.001, respectively; Fig. 2g, h). The 60-day OS rates for patients diagnosed during the first to sixth waves of COVID-19 were as follows: 38.9% (95% CI 17.5–60.0%; n = 18), 73.1% (95% CI 51.7–86.2%; n = 26), 86.7% (95% CI 77.3–92.4%; N = 83), 81.8% (95% CI 68.8–89.8%; n = 55), 95.7% (84.0–98.9; n = 47), and 94.1% (95% CI 88.6–97.0%; n = 138), respectively. Notably, the OS rates varied across the different epidemic waves, and higher mortality was observed during the early waves (details in Supplemental Fig. S3).

### Outcomes of complications during hospitalization

We also investigated several complications (thrombosis, hemorrhage, and new infections) associated with COVID-19 during hospitalization. The most common complications were additional infections, which occurred in 48 of 367 patients (13.1%), which were followed by thrombosis and bleeding (four and seven patients, respectively). The proportion of non-survivors was higher (35.4%) among patients who developed new infections during their hospital stays than among those who contracted new thrombosis (25.0%; 1/4) or hemorrhage (28.6%; 2/7; Supplemental Fig. S4a–c). Among all patients, the mortality rate was higher among patients with than without new infections (35.4% vs. 10.0%; P < 0.001).

## Discussion

Patients with HDs are at high risk of SARS-CoV-2 viral infection, because they require frequent hospitalization to receive treatment, including immunosuppressive therapies such as CD20 monoclonal antibodies, and have immune abnormalities caused by the HD itself. The timing of the COVID-19 outbreak and its strains vary among reports, but several studies have found that patients with HD have extremely poor COVID-19 outcomes, with mortality rates ranging from 28 to 61% [3–9]—values significantly higher than those in patients with COVID-19 in general. Notably, the mortality rate of patients with HDs in our study (13.3%), although lower than previously reported, was higher than that among patients registered in COVIREGI-JP (7.5%) [2]. The lower mortality rate for HDs in this study might have been due to differences in vaccination rates and advances in COVID-19 treatment. In fact, 30% of patients in our cohort had been immunized. Uchida et al. [12] have reported an extremely high mortality rate of patients with nosocomial infections; we also found a significantly higher mortality rate in patients with nosocomial infections (31.8%) than community-acquired infections (7.0%, P < 0.001). We concluded that the prognosis for patients with malignant HDs and nosocomial SARS-CoV-2 infection was extremely poor, possibly because most hospitalized patients who needed treatment for the disease or complications could not avoid the spread of the SARS-CoV-2 virus in the hospital and consequently were exposed to the virus for an extended period of time, as previously described [17].

The present study used two COVID-19 severity classifications for OS subgroup analysis: one from the ASH [4] and the other from the MHLW [15]. Both the ASH and MHLW severity classifications accurately predicted a decrease in OS with increasing severity at the time of COVID-19 diagnosis. According to both classifications, the 60-day OS rate for the severe group was approximately 60%, thus indicating their utility in identifying a subgroup of patients with particularly poor prognosis. In the multivariate analysis, the severe group, as determined by both severity classifications, was not found to be a factor contributing to OS. However, the need for oxygen at the time of diagnosis (HR 14.55), which is crucial in determining severity in both classifications, was identified as a significant adverse prognostic factor.

Several studies have reported the effects of patient condition (age, comorbidities, etc.), HD type, and current treatments on prognosis, but no conclusive risk factors have been identified. Age ≥ 60 years and recent systemic anticancer treatment have been reported as risk factors for death in a systematic review and meta-analysis of 3377 patients in Europe with COVID-19 and HDs, most of which were malignant [7]. A multicenter retrospective cohort study of patients in Italy with malignant HDs has also reported that older age, leukemia, plasma cell neoplasms, and lymphoma were all associated with reduced survival [8]. In addition to these disease types, progressive disease state and severe/critical SARS-CoV-2 infection status have been identified as significant factors. In contrast, two large studies on the prognosis of COVID-19-infected patients with all malignancies, including HDs, have found that a diagnosis of leukemia and lymphopenia/neutropenia in baseline laboratory data were the most significant prognostic factors [18, 19]. We performed both univariate and multivariate analyses combining the aforementioned characteristics (age, laboratory values, severity classification (ASH or MHLW), the HD itself, and disease condition, etc.). Older age or specific malignant HDs were not found to be prognostic factors, but Alb ≤ 3.3 g/dl and a need for oxygen were identified as unfavorable risk factors. The specific reasons underlying the association between hypoalbuminemia and poor prognosis remain unclear. However, our findings align with those from previous studies demonstrating a correlation between hypoalbuminemia and COVID-19 severity [20, 21]. To our knowledge, no reports have described the relationship between hypoalbuminemia and the prognosis of patients with COVID-19 with HDs. The pathophysiological mechanisms underlying hypoalbuminemia are generally attributed to various causes, including increased capillary permeability, decreased protein synthesis, and the presence of HDs themselves. Thus, the hypercytokinemia observed in patients with severe COVID-19 may contribute to hypoalbuminemia through the mechanisms described above [22].

Notably, our study revealed that patients with benign HDs had significantly better prognosis than patients with malignant HDs. Analyses limited to malignant HDs indicated that the HD itself was not a poor prognostic factor. ITP accounted for nearly half (20/43; 46.5%) of benign HD cases; three-quarters of these patients had high and/or low-moderate corticosteroids within 1 year before COVID-19 diagnosis, and 40% received low-moderate steroids at the time of diagnosis. Therefore, even if patients were undergoing immunosuppressive therapy, benign HDs might decrease the risk of mortality in patients with COVID-19.

This study had several limitations. First, its cross-sectional nature prevented detection of any detailed causal relationships between COVID-19 and death, and the relationships thus remain correlative. Furthermore, our study began later than previous studies, and background factors such as advances in therapeutic drugs against COVID-19 and an increase in vaccination rates in Japan might have differed with respect to earlier studies. In particular, new drugs that were not used in this cohort, such as the antiviral drugs molnupiravir [23] and nirmatrelvir plus ritonavir [24], and the neutralizing antibodies sotrovimab [25] and casirivimab plus imdevimab [26], have since been approved in Japan in a short time period. The role of anticoagulants (for example heparin) [27] in patients with severe disease has also been clarified. Our data also included information on the first to sixth epidemic waves of patients infected with various virus strains (first wave, B.1.1 strain; second wave, B.1.1.284 strain; third wave, B.1.1.214 strain; fourth wave, Alpha strain; fifth wave, Delta strain; and sixth wave, Omicron strain [15]). The emergence of new mutant strains is expected to alter the pathogenesis and fatality rate of COVID-19 in patients with HDs in the future. In addition, as described in the Results section, our study did not include all consecutive patients during the study period, thus potentially resulting in bias. Actually, 150 patients with a known prognosis were not enrolled in the study by May 2022.

Finally, the mortality rate of patients with HD hospitalized with COVID-19 in Japan was significantly lower than that reported in other countries. However, patients with more severe disease requiring oxygen at the time of diagnosis, and patients with comorbidities, particularly malignant HDs requiring antineoplastic agents, continue to face high mortality rates. Vaccination may be ineffective in these patients [28], and attempts have been made to prevent SARS-CoV-2 infection with antibodies such as tixagevimab–cilgavimab [29]. To further develop effective COVID-19 prevention and treatment methods in patients with HDs, continued research on SARS-CoV-2 infection is required, in light of the continuing threat of a pandemic caused by the Omicron strain in Japan.

### Supplementary Information

Below is the link to the electronic supplementary material.

Figure S1 Hematological diseases in all patients. Pie chart of composition of hematological diseases in all patients. AA, aplastic anemia; AML, acute myeloid leukemia; APL, acute promyelocytic leukemia; ATL(L), adult T-cell leukemia-(lymphoma); AUL, acute undifferentiated leukemia; CAD, cold agglutinin disease; CLL, chronic lymphocytic leukemia; CML, chronic myeloid leukemia; CMML, chronic myelomonocytic leukemia; ENTL, extranodal NK/T cell lymphoma; aggressive BL, aggressive B-cell lymphoma; aggressive TL, aggressive T-cell lymphoma; HL, Hodgkin lymphoma; IBL, indolent B-cell lymphoma; ITL, indolent T-cell lymphoma; ITP, idiopathic thrombocytopenic purpura; LPL, lymphoplasmacytic lymphoma; MCL, mantle cell lymphoma; MDS, myelodysplastic syndrome; MF, myelofibrosis; MM, multiple myeloma; MPAL, mixed-phenotype acute leukemia; MPN, myeloproliferative neoplasm; PCNSL, primary central nervous system lymphoma; Ph, Philadelphia; PNH, paroxysmal nocturnal hemoglobinuria; PRCA, pure red cell aplasia; ALL, acute lymphocytic leukemia; TTP, thrombotic thrombocytopenic purpura; wAIHA, warm autoimmune hemolytic anemia. Figure S2 Boxplots of laboratory test results at the time of COVID-19 diagnosis, stratified according to patient survival. WBC, white blood cell; Hb, hemoglobin; Plt, platelet; LDH, lactate dehydrogenase; Alb, albumin; AT, antithrombin; FDP, fibrin/fibrinogen degradation products; CRP, C-reactive protein; AST, aspartate aminotransferase; ALT, alanine aminotransferase. Figure S3 Survival in each epidemic wave of SARS-CoV-2 infection. Major strains of COVID-19 observed and defined period for each wave in this study are as follows. First wave (January 29, 2020, to June 13, 2020), European strain (B.1.1): second wave (June 14, 2020, to October 9, 2020), variant of European strain (B.1.1.284); third wave (October 10, 2020, to February 28, 2021), variant of European strain (B.1.1.214); fourth wave (March 1, 2021, to June 20, 2021), Alpha strain (B1.1.7); fifth wave (June 21, 2021, to December 16, 2021), Delta strain (AY.29); sixth wave (December 17, 2021, to June 24, 2022), Omicron strain (BA.1.1.2). Figure S4 Newly occurring events during hospitalization for SARS-CoV-2 infection. (a) Thrombotic, (b) bleeding, and (C) infectious disease events

Supplementary file2 (DOCX 89 KB)

## Data Availability

The authors confirm that the data supporting the findings of this study are available within the article [and/or] its supplementary materials.

## References

[1] https://data.who.int/dashboards/covid19/cases. Accessed 31 July 2023.

[2] Matsunaga N, Hayakawa K, Terada M, Ohtsu H, Asai Y, Tsuzuki S (2021). Clinical epidemiology of hospitalized patients with coronavirus disease 2019 (COVID-19) in Japan: report of the COVID-19 Registry Japan. Clin Infect Dis.

[3] He W, Chen L, Chen L, Yuan G, Fang Y, Chen W (2020). COVID-19 in persons with haematological cancers. Leukemia.

[4] Wood WA, Neuberg DS, Thompson JC, Tallman MS, Sekeres MA, Sehn LH (2020). Outcomes of patients with hematologic malignancies and COVID-19: a report from the ASH Research Collaborative Data Hub. Blood Adv.

[5] Malard F, Genthon A, Brissot E, van de Wyngaert Z, Marjanovic Z, Ikhlef S (2020). COVID-19 outcomes in patients with hematologic disease. Bone Marrow Transplant.

[6] Pagano L, Salmanton-García J, Marchesi F, Busca A, Corradini P, Hoenigl M, EPICOVIDEHA working group (2021). COVID-19 infection in adult patients with hematological malignancies: a European Hematology Association Survey (EPICOVIDEHA). J Hematol Oncol.

[7] Vijenthira A, Gong IY, Fox TA, Booth S, Cook G, Fattizzo B (2020). Outcomes of patients with hematologic malignancies and COVID-19: a systematic review and meta-analysis of 3377 patients. Blood.

[8] Passamonti F, Cattaneo C, Arcaini L, Bruna R, Cavo M, Merli F, ITA-HEMA-COV Investigators (2020). Clinical characteristics and risk factors associated with COVID-19 severity in patients with haematological malignancies in Italy: a retrospective, multicentre, cohort study. Lancet Haematol..

[9] Martín-Moro F, Marquet J, Piris M, Michael BM, Sáez AJ, Corona M (2020). Survival study of hospitalised patients with concurrent COVID-19 and haematological malignancies. Br J Haematol.

[10] Connors JM, Levy JH (2020). COVID-19 and its implications for thrombosis and anticoagulation. Blood.

[11] Horiuchi H, Morishita E, Urano T, Yokoyama K, Questionnaire-survey Joint Team on The COVID-19-related thrombosis (2021). COVID-19-related thrombosis in japan: final report of a questionnaire-based survey in 2020. J Atheroscler Thromb.

[12] Uchida T, Takagi Y, Mizuno A, Okamura H, Saito H, Ide S, et al. [Retrospective analysis of nosocomial COVID-19: a comparison between patients with hematological disorders and other diseases]. Rinsho Ketsueki. 2020;**61:**857–864. 10.11406/rinketsu.61.857 32908046

[13] http://www.jshem.or.jp/modules/research/index.php?content_id=2. Accessed 31 July 2023.

[14] https://center6.umin.ac.jp/cgi-open-bin/ctr/ctr_view.cgi?recptno=R000049979. Accessed 31 July 2023.

[15] Clinical Guide Version 9.0 (From ministry of health, labour and welfare (MHLW)) https://www.mhlw.go.jp/content/000936655.pdf. Accessed 31 July 2023.

[16] Kanda Y (2013). Investigation of the freely available easy-to-use software 'EZR' for medical statistics. Bone Marrow Transplant.

[17] Aydillo T, Gonzalez-Reiche AS, Aslam S, van de Guchte A, Khan Z, Obla A (2020). Shedding of viable SARS-CoV-2 after immunosuppressive therapy for cancer. N Engl J Med.

[18] Lee LYW, Cazier JB, Starkey T, Briggs SEW, Arnold R, Bisht V, UK Coronavirus Cancer Monitoring Project Team (2020). COVID-19 prevalence and mortality in patients with cancer and the effect of primary tumour subtype and patient demographics: a prospective cohort study. Lancet Oncol.

[19] Jee J, Foote MB, Lumish M, Stonestrom AJ, Wills B, Narendra V (2020). Chemotherapy and COVID-19 outcomes in patients with cancer. J Clin Oncol.

[20] Aziz M, Fatima R, Lee-Smith W, Assaly R (2020). The association of low serum albumin level with severe COVID-19: a systematic review and meta-analysis. Crit Care.

[21] Kheir M, Saleem F, Wang C, Mann A, Chua J (2021). Higher albumin levels on admission predict better prognosis in patients with confirmed COVID-19. PLoS ONE.

[22] Hu B, Huang S, Yin L (2021). The cytokine storm and COVID-19. J Med Virol.

[23] Jayk Bernal A, Gomes da Silva MM, Musungaie DB, Kovalchuk E, Gonzalez A, Delos Reyes V, MOVe-OUT Study Group (2022). Molnupiravir for oral treatment of covid-19 in nonhospitalized patients. N Engl J Med.

[24] Hammond J, Leister-Tebbe H, Gardner A, Abreu P, Bao W, Wisemandle W, EPIC-HR Investigators (2022). Oral nirmatrelvir for high-risk, nonhospitalized adults with Covid-19. N Engl J Med.

[25] Gupta A, Gonzalez-Rojas Y, Juarez E, Crespo Casal M, Moya J, Falci DR, COMET-ICE Investigators (2021). Early treatment for Covid-19 with SARS-CoV-2 neutralizing antibody sotrovimab. N Engl J Med.

[26] O'Brien MP, Forleo-Neto E, Sarkar N, Isa F, Hou P, Chan KC, COVID-19 Phase 3 Prevention Trial Team (2022). Effect of subcutaneous casirivimab and imdevimab antibody combination vs placebo on development of symptomatic COVID-19 in early asymptomatic SARS-CoV-2 infection: a randomized clinical trial. JAMA.

[27] Sholzberg M, Tang GH, Rahhal H, AlHamzah M, Kreuziger LB, Áinle FN, RAPID trial investigators (2021). Effectiveness of therapeutic heparin versus prophylactic heparin on death, mechanical ventilation, or intensive care unit admission in moderately ill patients with covid-19 admitted to hospital: RAPID randomised clinical trial. BMJ.

[28] Narita K, Nakaji S, Tabata R, Terao T, Kuzume A, Tsushima T (2022). Antibody response to COVID-19 vaccination in patients with lymphoma. Int J Hematol.

[29] Levin MJ, Ustianowski A, De Wit S, Launay O, Avila M, Templeton A, PROVENT Study Group (2022). Intramuscular AZD7442 (Tixagevimab-Cilgavimab) for Prevention of Covid-19. N Engl J Med.

