# Supplementary material for: Characteristics and prognosis of patients with COVID-19 and hematological diseases in Japan: a cross-sectional study
Source: Int J Hematol. 2024 Jan 3;119(2):183–95. doi: 10.1007/s12185-023-03685-w (PMC10830869; doi:10.1007/s12185-023-03685-w)

Supplemental Figure S1

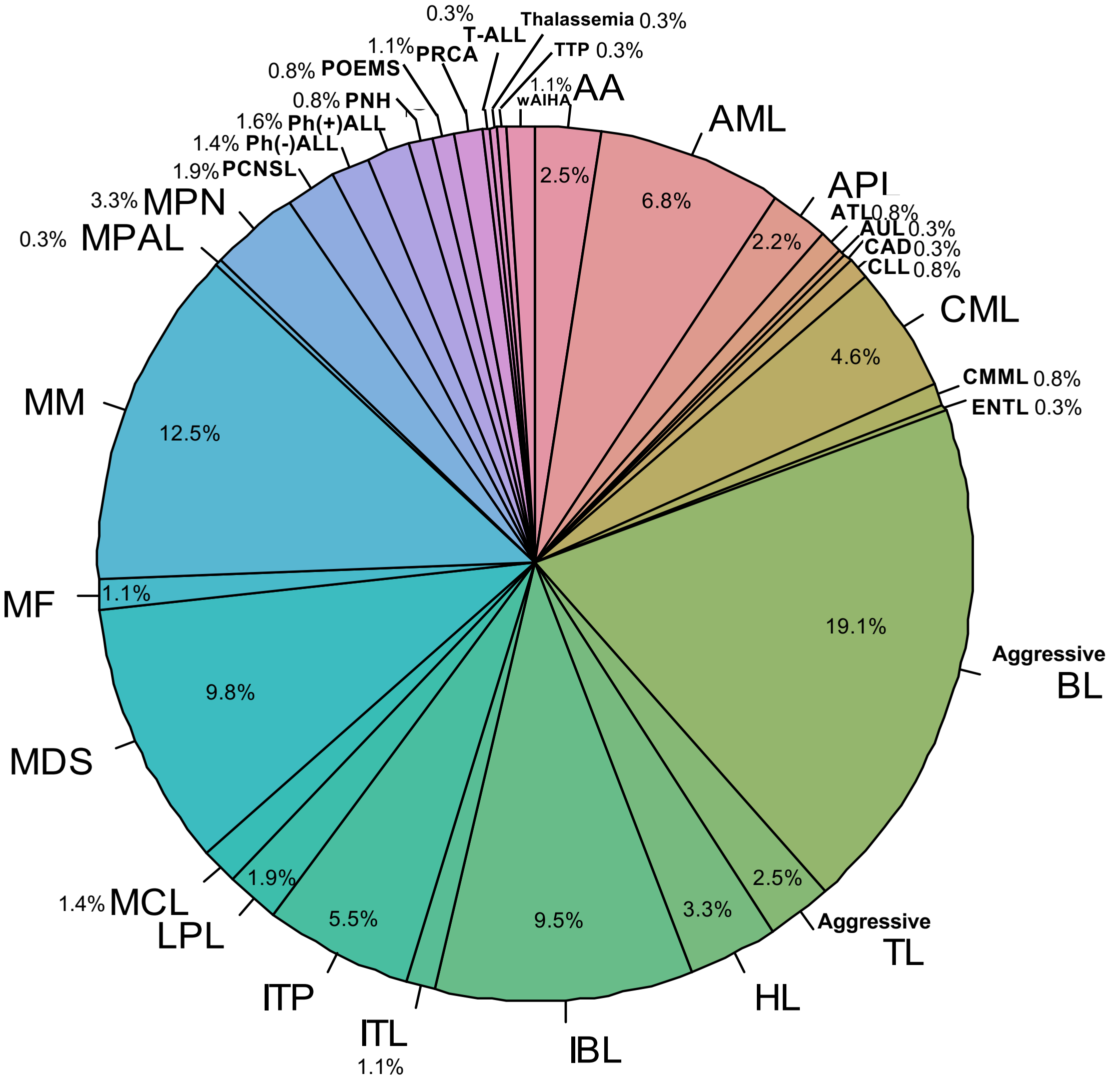

Supplemental Figure S2

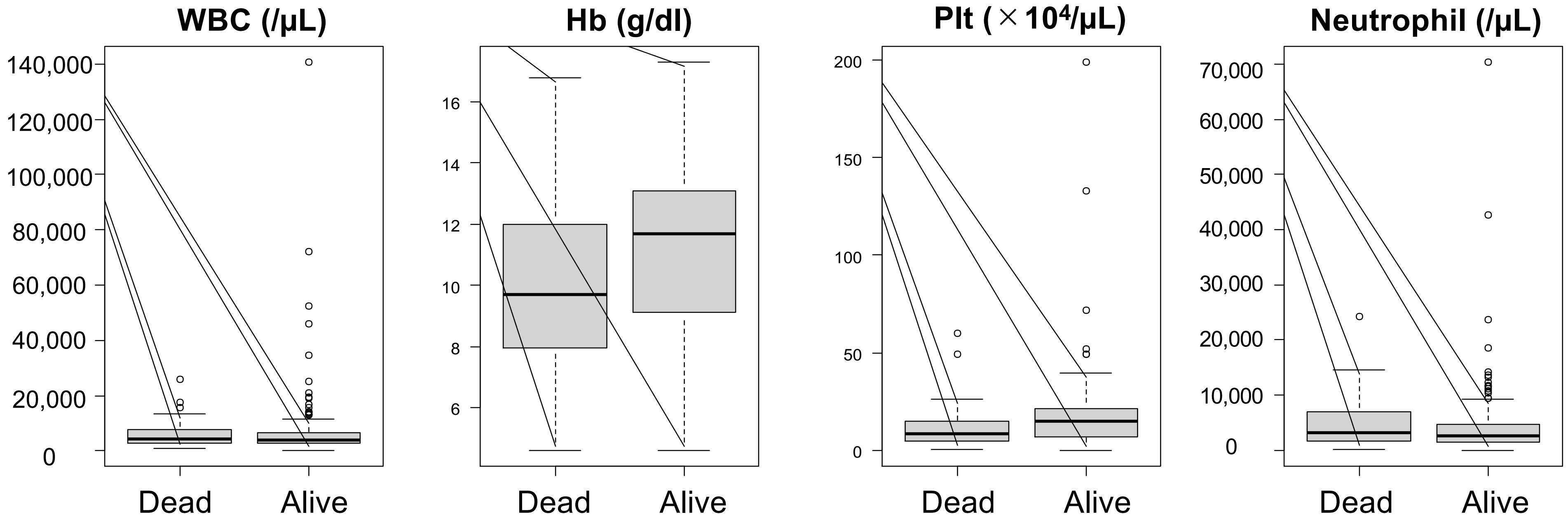

5984 vs. 6252 (P=0.856)

10.0 vs. 11.2 (P=0.005)

11.4 vs. 16.1 (P=0.068)

4597 vs. 2988 (P=0.502)

Supplemental Figure S2

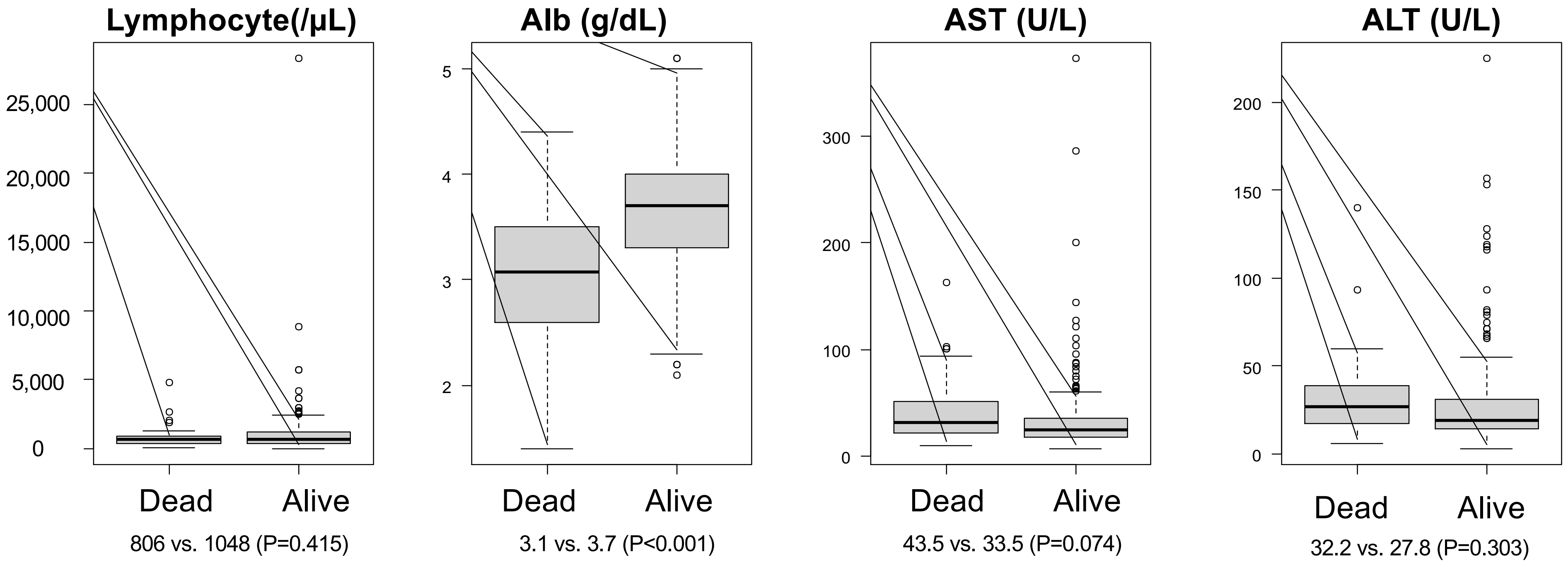

Supplemental Figure S2

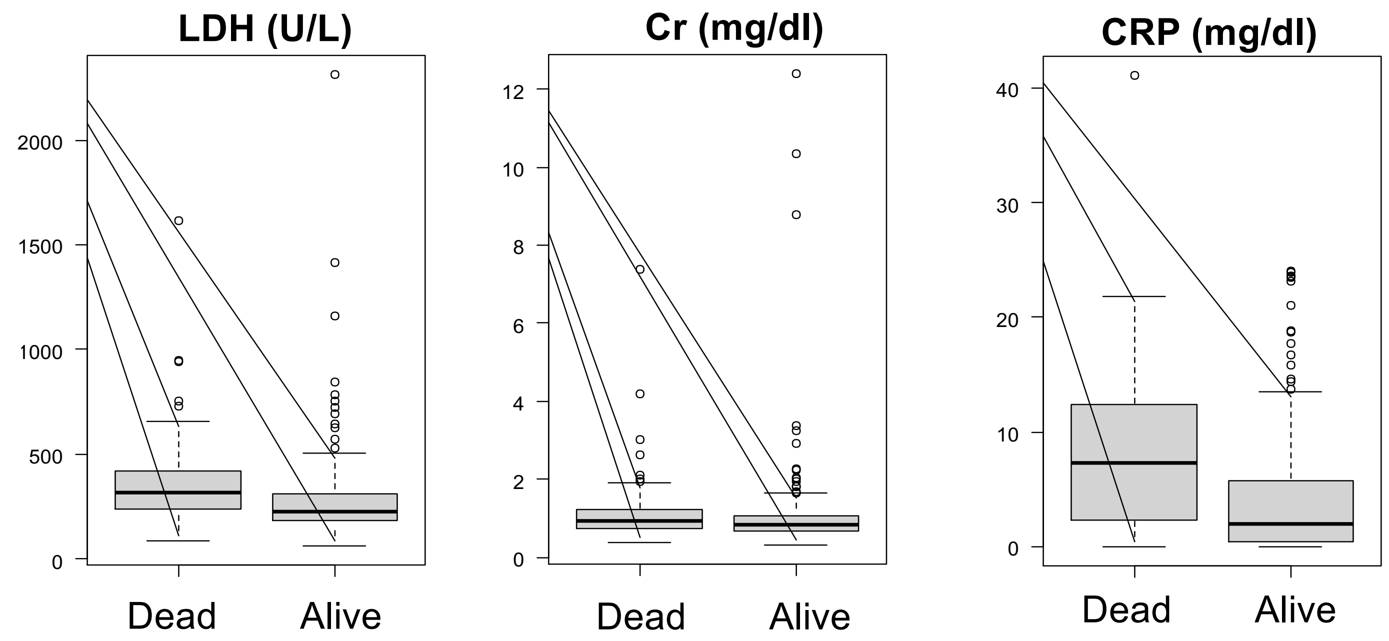

380 vs. 272 (P=0.001)

1.28 vs. 1.03 (P=0.188)

8.8 vs. 4.1 (P<0.001)

Supplemental Figure S2

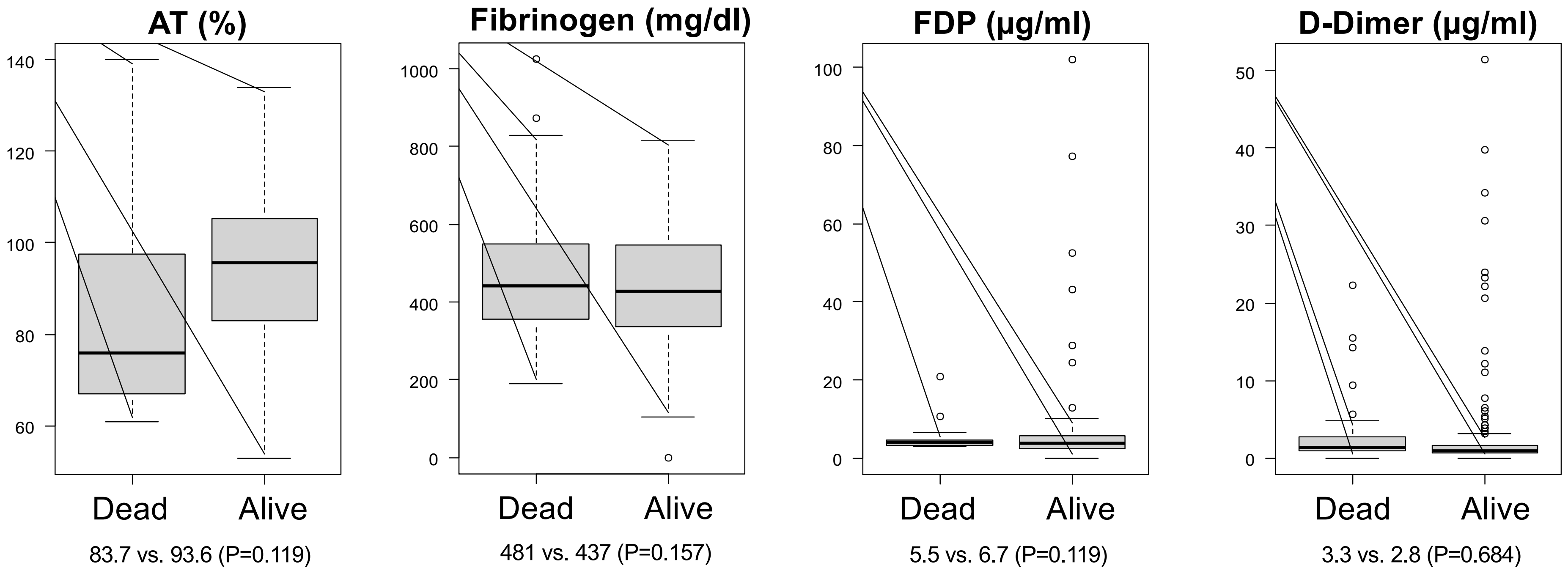

Supplemental Figure S3

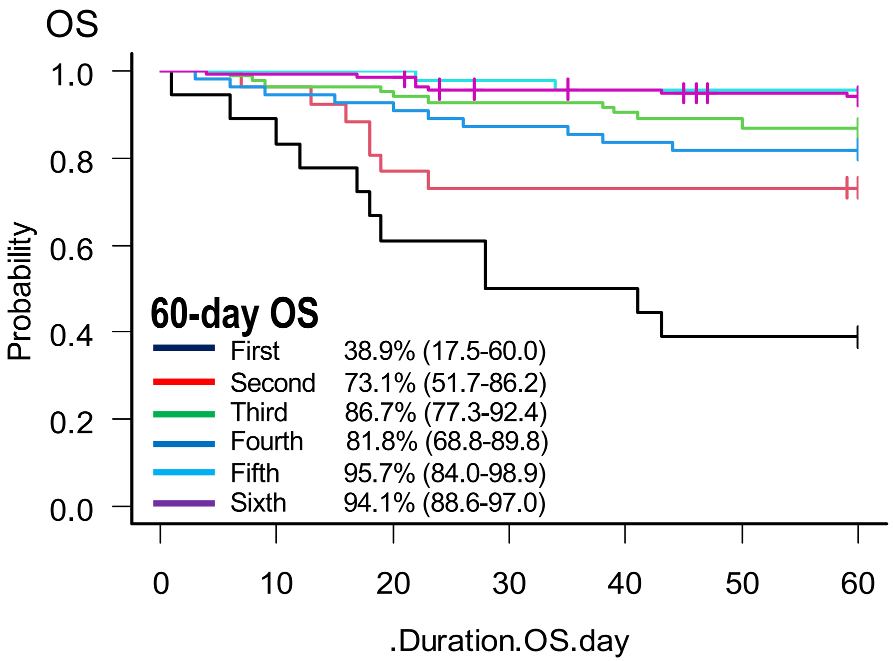

Number at risk

18	16	11	9	9	7	7
26	25	20	19	19	19	18
83	80	79	77	75	74	72
55	52	51	48	46	45	45
47	47	47	46	45	45	45
138	137	136	129	128	124	123

Supplemental Figure S4

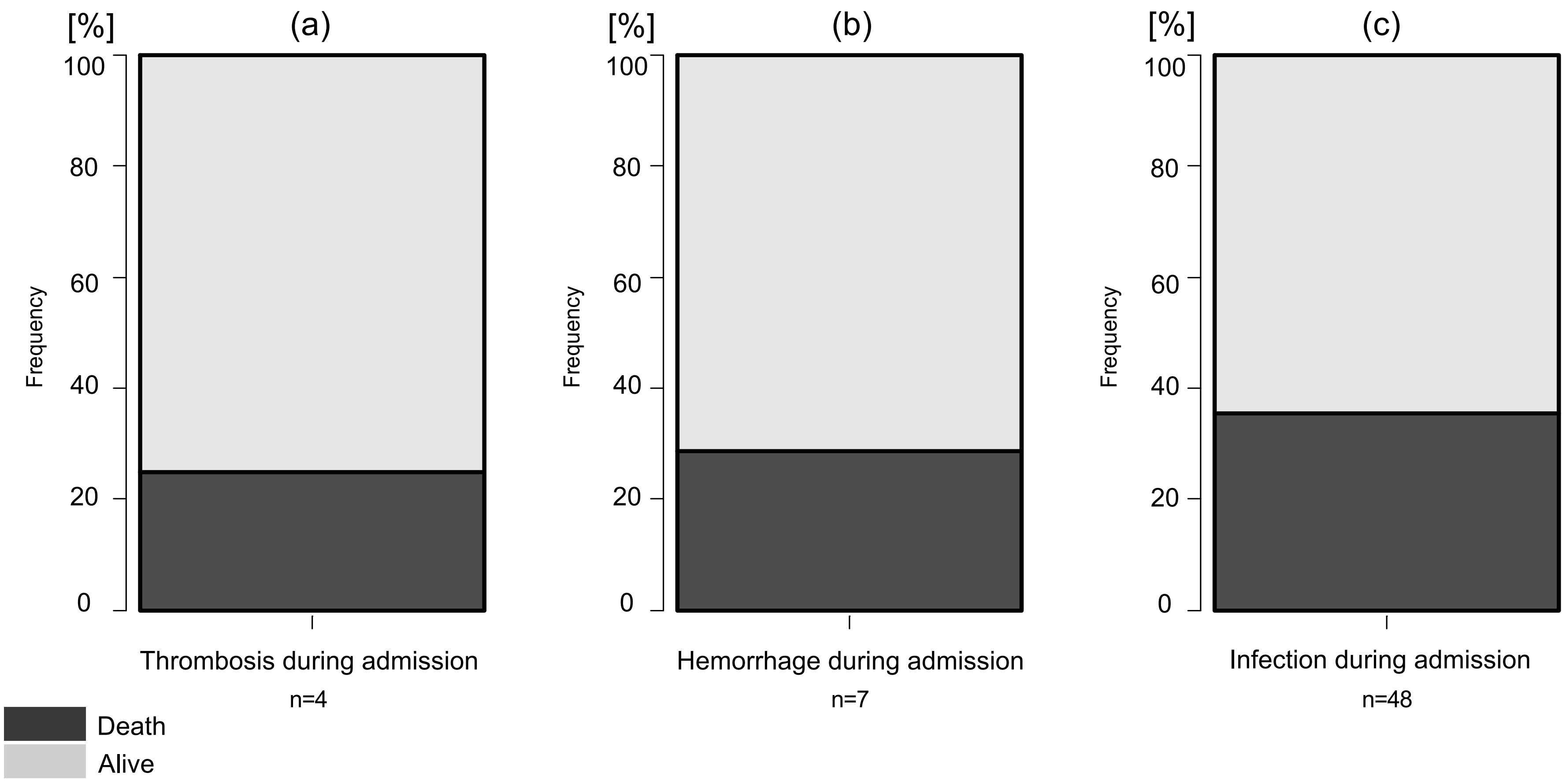

Supplement: Supplementary file 1 — Figure S1 Hematological diseases in all patients. Pie chart of composition of hematological diseases in all patients. AA, aplastic anemia; AML, acute myeloid leukemia; APL, acute promyelocytic leukemia; ATL(L), adult T-cell leukemia-(lymphoma); AUL, acute undifferentiated leukemia; CAD, cold agglutinin disease; CLL, chronic lymphocytic leukemia; CML, chronic myeloid leukemia; CMML, chronic myelomonocytic leukemia; ENTL, extranodal NK/T cell lymphoma; aggressive BL, aggressive B-cell lymphoma; aggressive TL, aggressive T-cell lymphoma; HL, Hodgkin lymphoma; IBL, indolent B-cell lymphoma; ITL, indolent T-cell lymphoma; ITP, idiopathic thrombocytopenic purpura; LPL, lymphoplasmacytic lymphoma; MCL, mantle cell lymphoma; MDS, myelodysplastic syndrome; MF, myelofibrosis; MM, multiple myeloma; MPAL, mixed-phenotype acute leukemia; MPN, myeloproliferative neoplasm; PCNSL, primary central nervous system lymphoma; Ph, Philadelphia; PNH, paroxysmal nocturnal hemoglobinuria; PRCA, pure red cell aplasia; ALL, acute lymphocytic leukemia; TTP, thrombotic thrombocytopenic purpura; wAIHA, warm autoimmune hemolytic anemia. Figure S2 Boxplots of laboratory test results at the time of COVID-19 diagnosis, stratified according to patient survival. WBC, white blood cell; Hb, hemoglobin; Plt, platelet; LDH, lactate dehydrogenase; Alb, albumin; AT, antithrombin; FDP, fibrin/fibrinogen degradation products; CRP, C-reactive protein; AST, aspartate aminotransferase; ALT, alanine aminotransferase. Figure S3 Survival in each epidemic wave of SARS-CoV-2 infection. Major strains of COVID-19 observed and defined period for each wave in this study are as follows. First wave (January 29, 2020, to June 13, 2020), European strain (B.1.1): second wave (June 14, 2020, to October 9, 2020), variant of European strain (B.1.1.284); third wave (October 10, 2020, to February 28, 2021), variant of European strain (B.1.1.214); fourth wave (March 1, 2021, to June 20, 2021), Alpha strain (B1.1.7); fifth wave (Jun [file 12185_2023_3685_MOESM1_ESM.pdf]
