# Supplementary material for: Characteristics and prognosis of patients with COVID-19 and hematological diseases in Japan: a cross-sectional study
Source: Int J Hematol. 2024 Jan 3;119(2):183–95. doi: 10.1007/s12185-023-03685-w (PMC10830869; doi:10.1007/s12185-023-03685-w)
Supplement: Supplementary file 2 — Supplementary file2 (DOCX 89 KB) [file 12185_2023_3685_MOESM2_ESM.docx]

**Supplemental Table**

**Table S1 Laboratory and imaging data at COVID-19 diagnosis**

| Laboratory data | | Imaging data | |
| --- | --- | --- | --- |
| WBC, median (range), /µL (n=297) | 4,200 (200–140,700) | X-ray | |
| Neutrophils, median (range), /µL (n=294) | 2,816 (0–70,350) | Normal/abnormal, n (%) | 134 (36.5%)/111 (30.3%) |
| Lymphocytes, median (range), /µL (n=295) | 688 (0–28,372) | -Unilateral/bilateral (n=111), n (%) | 17 (15.3%)/94 (84.7%) |
| Hemoglobin, median (range), g/dL (n=297) | 11.3 (4.6–17.3) | -Consolidation (n=111), n (%) | 25 (22.5%) |
| Platelets, median (range), *10^4^/µL (n=297) | 13.2 (0.1–198.8) | -GGO or GGA (n=111), n (%) | 81 (73.0%) |
| Lactate dehydrogenase, median (range), U/L (n=296) | 233 (63–2,313) | -Both (n=111), n (%) | 5 (4.5%) |
| Albumin, median (range), g/dL (n=283) | 3.6 (1.4–5.1) | Others/unknown, n (%) | 22 (6.0%)/100 (27.3%) |
| Fibrinogen, median (range), mg/dL (n=188) | 431.5 (104–1,025) | CT | |
| Antithrombin, median (range), % (n=57) | 95 (53–140) | Normal/abnormal, n (%) | 62 (16.9%)/166 (45.2%) |
| FDP, median (range), µg/mL (n=124) | 3.8 (0–102) | -Unilateral/bilateral (n=166), n (%) | 24 (14.5%)/142 (85.5%) |
| D-dimer, median (range), µg/mL (n=203) | 1 (0–51.4) | -Consolidation (n=166), n (%) | 16 (9.6%) |
| CRP, median (range), mg/dL (n=293) | 2.36 (0–41.1) | -GGO or GGA (n=166), n (%) | 136 (81.9%) |
| AST, median (range), U/L (n=293) | 25 (7–373) | -Both (N=166), n (%) | 14 (8.4%) |
| ALT, median (range), U/L (n=294) | 19.5 (3–225) | Others/unknown, n (%) | 23 (6.3)/116 (31.6%) |
| Creatinine, median (range), mg/dL (n=293) | 0.84 (0.33–12.39) |  |  |

WBC, white blood cell; FDP, fibrin/fibrinogen degradation products; CRP, C-reactive protein; AST, aspartate aminotransferase; ALT, alanine aminotransferase; CT, computed tomography; GGO, ground-glass opacity; GGA, ground-glass attenuation.

**Table S2 (1)-(33) Ongoing treatments for hematological disease or treatments given within the 1 year before COVID-19 diagnosis.**

**(1) AA n=9**

| **Variable** | **Patients (n=9)** |
| --- | --- |
| **Treatments for hematological disease within the last year** | |
| No treatment, observation, n (%) | 0 (0.0) |
| Transfusion RBC / PC, n (%) | 3 (33.3) / 2 (22.2) |
| G-CSF, n (%) | 1 (11.1) |
| Iron chelation, n (%) | 2 (22.2) |
| High dose steroid, n (%) | 0 (0.0) |
| Low-moderate steroid, n (%) | 0 (0.0) |
| CyA, n (%) | 9 (100) |
| ATG, n (%) | 2 (22.2) |
| Eltrombopag, n (%) | 7 (77.8) |
| Romiplostim, n (%) | 1 (11.1) |
| Others, n(%) | 0 (0.0) |
| **Ongoing treatments for hematological disease at COVID-19 Dx** | |
| No treatment, observation, n (%) | 1 (11.1) |
| Transfusion RBC / PC, n (%) | 1 (11.1) / 1(11.1) |
| G-CSF, n (%) | 0 (0.0) |
| Iron chelation, n (%) | 2 (22.2) |
| High dose steroid, n (%) | 0 (0.0) |
| Low-moderate steroid, n (%) | 0 (0.0) |
| CyA, n (%) | 8 (88.9) |
| ATG, n (%) | 0 (0.0) |
| Eltrombopag, n (%) | 6 (66.7) |
| Romiplostim, n (%) | 1 (11.1) |
| Others, n (%) | 0 (0.0) |

**(2) AML n=25**

| **Variable** | **Patients (n=25)** |
| --- | --- |
| **Treatments for hematological disease within the last year** | |
| No treatment, observation, n (%) | 12 (48.0) |
| Intensive induction therapy, n (%) | 8 (32.0) |
| Consolidation therapy, n (%) | 7 (28.0) |
| Maintenance therapy, n (%) | 0 (0.0) |
| Low dose palliative therapy, n (%) | 5 (20.0) |
| Azacitidine, n (%) | 5 (20.0) |
| Gilteritinib, n (%) | 2 (8.0) |
| Quizartinib, n (%) | 1 (4.0) |
| Venetoclax, n (%) | 1 (4.0) |
| Others, n (%) | 1 (4.0) |
| **Ongoing treatments for hematological disease at COVID-19 Dx** | |
| No treatment, observation, n (%) | 14 (56.0) |
| Intensive induction therapy, n (%) | 0 (0.0) |
| Consolidation therapy, n (%) | 4 (16.0) |
| Maintenance therapy, n (%) | 0 (0.0) |
| Low dose palliative therapy, n (%) | 2 (8.0) |
| Azacitidine, n (%) | 3 (12.0) |
| Gilteritinib, n (%) | 2 (8.0) |
| Quizartinib, n (%) | 1 (4.0) |
| Venetoclax, n (%) | 1 (4.0) |
| Others, n (%) | 1 (4.0) |

**(3) APL n＝8**

| **Variable** | **Patients (n=8)** |
| --- | --- |
| **Treatments for hematological disease within the last year** | |
| No treatment, observation, n (%) | 3 (37.5) |
| Intensive induction therapy, n (%) | 4 (50.0) |
| Consolidation therapy, n (%) | 5 (62.5) |
| Maintenance therapy, n (%) | 2 (25.0) |
| ATRA, n (%) | 4 (50.0) |
| Tamibarotene, n (%) | 1 (12.5) |
| ATO, n (%) | 2 (25.0) |
| Gemtuzumab ozogamicin, n (%) | 5 (62.5) |
| Others, n (%) | 0 (0.0) |
| **Ongoing treatments for hematological disease at COVID-19 Dx** | |
| No treatment, observation, n (%) | 3 (37.5) |
| Intensive induction therapy, n (%) | 0 (0.0) |
| Consolidation therapy, n (%) | 2 (25.0) |
| Maintenance therapy, n (%) | 1 (12.5) |
| ATRA, n (%) | 1 (12.5) |
| Tamibarotene, n (%) | 1 (12.5) |
| ATO, n (%) | 1 (12.5) |
| Gemtuzumab ozogamicin, n (%) | 0 (0.0) |
| Others, n (%) | 0 (0.0) |

**(4) ATL n=3**

| **Variable** | **Patients (n=3)** |
| --- | --- |
| **Treatments for hematological disease within the last year** | |
| No treatment, observation, n (%) | 1 (33.3) |
| High dose steroid, n (%) | 1 (33.3) |
| Low-moderate steroid, n (%) | 1 (33.3) |
| Anthracycline-based chemotherapy, n (%) | 2 (66.7) |
| Modified LSG15 therapy, n (%) | 0 (0.0) |
| Pentostatin, n (%) | 0 (0.0) |
| Sobuzoxane, n (%) | 0 (0.0) |
| Mogamulizumab, n (%) | 0 (0.0) |
| Lenalidomide, n (%) | 0 (0.0) |
| Radiation, n (%) | 0 (0.0) |
| Others, n (%) | 1 (33.3) |
| **Ongoing treatments for hematological disease at COVID-19 Dx** | |
| No treatment, observation, n (%) | 1 (33.3) |
| High dose steroid, n (%) | 0 (0.0) |
| Low-moderate steroid, n (%) | 1 (33.3) |
| Anthracycline-based chemotherapy, n (%) | 1 (33.3) |
| Modified LSG15 therapy, n (%) | 0 (0.0) |
| Pentostatin, n (%) | 0 (0.0) |
| Sobuzoxane, n (%) | 0 (0.0) |
| Mogamulizumab, n (%) | 0 (0.0) |
| Lenalidomide, n (%) | 0 (0.0) |
| Radiation, n (%) | 0 (0.0) |
| Others, n (%) | 1 (33.3) |

**(5) AUL n=1**

| **Variable** | **Patients (n=1)** |
| --- | --- |
| **Treatments for hematological disease within the last year** | |
| No treatment, observation, n (%) | 0 (0.0) |
| Intensive induction therapy, n (%) | 1 (100.0) |
| Consolidation therapy, n (%) | 1 (100.0) |
| Maintenance therapy, n (%) | 0 (0.0) |
| Low dose palliative therapy, n (%) | 0 (0.0) |
| Gilteritinib, n (%) | 0 (0.0) |
| Quizartinib, n (%) | 0 (0.0) |
| Others, n (%) | 0 (0.0) |
| **Ongoing treatments for hematological disease at COVID-19 Dx** | |
| No treatment, observation, n (%) | 0 (0.0) |
| Intensive induction therapy, n (%) | 1 (100.0) |
| Consolidation therapy, n (%) | 0 (0.0) |
| Maintenance therapy, n (%) | 0 (0.0) |
| Low dose palliative therapy, n (%) | 0 (0.0) |
| Gilteritinib, n (%) | 0 (0.0) |
| Quizartinib, n (%) | 0 (0.0) |
| Others, n (%) | 0 (0.0) |

**(6) CAD n=1**

| **Variable** | **Patients (n=1)** |
| --- | --- |
| **Treatments for hematological disease within the last year** | |
| No treatment, observation, n (%) | 0 (0.0) |
| Keep warm, n (%) | 1 (100) |
| Infusion RBC, n (%) | 1 (100) |
| High dose steroid, n (%) | 0 (0.0) |
| Low-moderate steroid, n (%) | 0 (0.0) |
| Splenectomy, n (%) | 0 (0.0) |
| Others, n (%) | 0 (0.0) |
| **Ongoing treatments for hematological disease at COVID-19 Dx** | |
| No treatment, observation, n (%) | 0 (0.0) |
| Keep warm, n (%) | 1 (100) |
| Infusion RBC, n (%) | 1 (100) |
| High dose steroid, n (%) | 0 (0.0) |
| Low-moderate steroid, n (%) | 0 (0.0) |
| Splenectomy, n (%) | 0 (0.0) |
| Others, n (%) | 0 (0.0) |

**(7) CLL/SLL n=3**

| **Variable** | **Patients (n=3)** |
| --- | --- |
| **Treatments for hematological disease within the last year** | |
| No treatment, observation, n (%) | 2 (66.7) |
| Fludarabine based regimen, n (%) | 0 (0.0) |
| Bendamustine, n (%) | 0 (0.0) |
| Cyclophosphamide, n (%) | 0 (0.0) |
| Rituximab, n (%) | 0 (0.0) |
| Ofatumumab, n (%) | 0 (0.0) |
| Alemtuzumab, n (%) | 0 (0.0) |
| Ibrutinib, n (%) | 1 (33.3) |
| Venetoclax, n (%) | 0 (0.0) |
| Others, n (%) | 0 (0.0) |
| **Ongoing treatments for hematological disease at COVID-19 Dx** | |
| No treatment, observation, n (%) | 2 (66.7) |
| Fludarabine based regimen, n (%) | 0 (0.0) |
| Bendamustine, n (%) | 0 (0.0) |
| Cyclophosphamide, n (%) | 0 (0.0) |
| Rituximab, n (%) | 0 (0.0) |
| Ofatumumab, n (%) | 0 (0.0) |
| Alemtuzumab, n (%) | 0 (0.0) |
| Ibrutinib, n (%) | 1 (33.3) |
| Venetoclax, n (%) | 0 (0.0) |
| Others, n (%) | 0 (0.0) |

**(8) CML n=17**

| **Variable** | **Patients (n=17)** |
| --- | --- |
| **Treatments for hematological disease within the last year** | |
| No treatment, observation, n (%) | 4 (23.5) |
| Imatinib, n (%) | 3 (17.7) |
| Dasatinib, n (%) | 8 (47.1) |
| Nilotinib, n (%) | 1 (5.9) |
| Bosutinib, n (%) | 2 (11.8) |
| Ponatinib, n (%) | 2 (11.8) |
| Unknown, n (%) | 1 (5.9) |
| Others, n (%) | 0 (0.0) |
| **Ongoing treatments for hematological disease at COVID-19 Dx** | |
| No treatment, observation, n (%) | 6 (35.3) |
| Imatinib, n (%) | 2 (11.8) |
| Dasatinib, n (%) | 6 (35.3) |
| Nilotinib, n (%) | 0 (0.0) |
| Bostinib, n (%) | 1 (5.9) |
| Ponatinib, n (%) | 2 (11.8) |
| Others, n (%) | 0 (0.0) |

**(9) CMML n=3**

| **Variable** | **Patients (n=3)** |
| --- | --- |
| **Treatments for hematological disease within the last year** | |
| No treatment, observation, n (%) | 1 (33.3) |
| Transfusion RBC / PC, n (%) | 1 (33.3) / 0 (0.0) |
| Iron chelation, n (%) | 0 (0.0) |
| Hydroxyurea, n (%) | 0 (0.0) |
| Azacitidine, n (%) | 2 (66.7) |
| Others, n (%) | 0 (0.0) |
| **Ongoing treatments for hematological disease at COVID-19 Dx** | |
| No treatment, observation, n (%) | 1 (33.3) |
| Transfusion RBC / PC, n (%) | 1 (33.3) / 0 (0.0) |
| Iron chelation, n (%) | 0 (0.0) |
| Hydroxyurea, n (%) | 0 (0.0) |
| Azacitidine, n (%) | 2 (66.7) |
| Others, n (%) | 0 (0.0) |

**(10) ENTL n=1**

| **Variable** | **Patients (n=1)** |
| --- | --- |
| **Treatments for hematological disease within the last year** | |
| No treatment, observation, n (%) | 1 (0.0) |
| 2/3 DEVIC , n (%) | 0 (0.0) |
| SMILE , n (%) | 0 (0.0) |
| Radiation, n (%) | 0 (0.0) |
| Others, n (%) | 0 (0.0) |
| **Ongoing treatments for hematological disease at COVID-19 Dx** | |
| No treatment, observation, n (%) | 1 (0.0) |
| 2/3 DEVIC, n (%) | 0 (0.0) |
| SMILE, n (%) | 0 (0.0) |
| Radiation, n (%) | 0 (0.0) |
| Others, n (%) | 0 (0.0) |

**(11) Aggressive BL n=70**

| **Variable** | **Patients (n=70)** |
| --- | --- |
| **Treatments for hematological disease within the last year** | |
| No treatment, observation, n (%) | 21 (30.0) |
| High dose steroid, n (%) | 4 (5.7) |
| Low-moderate dose steroid, n (%) | 22 (31.4) |
| Anthracycline-based Chemotherapy, n (%) | 39 (55.7) |
| Bendamustine, n (%) | 1 (1.4) |
| HyperCVAD/MA, n (%) | 1 (1.4) |
| High dose AraC-based therapy, n (%) | 7 (10.0) |
| CODOX-M/IVAC, n (%) | 0 (0.0) |
| Platinum based chemotherapy, n (%) | 12 (17.1) |
| Rituximab, n (%) | 44 (62.9) |
| Polatuzumab vedotin, n (%) | 1 (1.4) |
| CD19-CAR T, n (%) | 0 (0.0) |
| Radiation / Operation, n (%) | 6 (8.6) / 3 (4.3) |
| Others, n (%) | 3 (4.3) |
| **Ongoing treatments for hematological disease at COVID-19 Dx** | |
| No treatment, observation, n (%) | 32 (45.7) |
| High dose steroid, n (%) | 1 (1.4) |
| Low-moderate dose steroid, n (%) | 9 (12.9) |
| Anthracycline-based Chemotherapy, n (%) | 22 (31.4) |
| Bendamustine, n (%) | 1 (1.4) |
| HyperCVAD/MA, n (%) | 0 (0.0) |
| High dose AraC-based chemotherapy, n (%) | 3 (4.3) |
| CODOX-M/IVAC, n (%) | 0 (0.0) |
| Platinum based chemotherapy, n (%) | 6 (8.6) |
| Rituximab, n (%) | 24 (34.3) |
| Polatuzumab vedotin, n (%) | 1 (1.4) |
| CD19-CAR T, n (%) | 0 (0.0) |
| Radiation / Operation, n (%) | 3 (4.3) / 0 (0.0) |
| Others, n (%) | 2 (2.9) |

**(12) Aggressive TL n=9**

| **Variable** | **Patients (n=9)** |
| --- | --- |
| **Treatments for hematological disease within the last year** | |
| No treatment, observation, n (%) | 3 (33.3) |
| High dose steroid, n (%) | 0 (0.0) |
| Low-moderate dose steroid, n (%) | 1 (11.1) |
| Anthracycline-based Chemotherapy, n (%) | 3 (33.3) |
| Brentuximab vedotin, n (%) | 2 (22.2) |
| Mogamulizumab, n (%) | 0 (0.0) |
| Pralatrexate, n (%) | 1 (11.1) |
| Romidepsin, n (%) | 0 (0.0) |
| Vorinostat, n (%) | 0 (0.0) |
| Forodesin, n (%) | 0 (0.0) |
| Radiation, n (%) | 1 (11.1) |
| Others, n (%) | 3 (33.3) |
| **Ongoing treatments for hematological disease at COVID-19 Dx** | |
| No treatment, observation, n (%) | 6 (66.7) |
| High dose steroid, n (%) | 0 (0.0) |
| Low-moderate dose steroid, n (%) | 0 (0.0) |
| Anthracycline-based Chemotherapy, n (%) | 0 (0.0) |
| Brentuximab vedotin, n (%) | 1 (11.1) |
| Mogamulizumab, n (%) | 0 (0.0) |
| Pralatrexate, n (%) | 0 (0.0) |
| Romidepsin, n (%) | 0 (0.0) |
| Vorinostat, n (%) | 0 (0.0) |
| Forodesin, n (%) | 0 (0.0) |
| Radiation, n (%) | 0 (0.0) |
| Others, n (%) | 2 (22.2) |

**(13) HL n=12**

| **Variable** | **Patients (n=12)** |
| --- | --- |
| **Treatments for hematological disease within the last year** | |
| No treatment, observation, n (%) | 4 (33.3) |
| ABVD, n (%) | 5 (41.7) |
| AVD, n (%) | 2 (16.7) |
| Platinum based chemotherapy, n (%) | 0 (0.0) |
| Brentuximab vedotin, n (%) | 4 (33.3) |
| Nivolumab, n (%) | 0 (0.0) |
| Pembrolizumab, n (%) | 0 (0.0) |
| Others, n (%) | 0 (0.0) |
| **Ongoing treatments for hematological disease at COVID-19 Dx** | |
| No treatment, observation, n (%) | 8 (66.7) |
| ABVD, n (%) | 2 (16.7) |
| AVD, n (%) | 1 (8.3) |
| Platinum based chemotherapy, n (%) | 0 (0.0) |
| Brentuximab vedotin, n (%) | 2 (16.7) |
| Nivolumab, n (%) | 0 (0.0) |
| Pembrolizumab, n (%) | 0 (0.0) |
| Others, n (%) | 0 (0.0) |

**(14) IBL n=35**

| **Variable** | **Patients (n=35)** |
| --- | --- |
| **Treatments for hematological disease within the last year** | |
| No treatment, observation, n (%) | 11 (31.4) |
| High dose steroid, n (%) | 2 (5.7) |
| Low-moderate dose steroid, n (%) | 3 (8.6) |
| Bendamustine, n (%) | 10 (28.6) |
| Fludarabine, n (%) | 0 (0.0) |
| Cladribine, n (%) | 0 (0.0) |
| Anthracycline-based Chemotherapy, n (%) | 4 (11.4) |
| Rituximab, n (%) | 12 (34.3) |
| Obinutuzumab, n (%) | 8 (22.9) |
| Ibritumomab tiuxetan, n (%) | 0 (0.0) |
| Lenalidomide, n (%) | 1 (2.9) |
| Radiation / Operation, n (%) | 3 (8.6) / 1 (2.9) |
| Eradication Tx for Helicobacter. / Other, n (%) | 0 (0.0) / 3 (8.6) |
| **Ongoing treatments for hematological disease at COVID-19 Dx** | |
| No treatment, observation, n (%) | 18 (51.4) |
| High dose steroid, n (%) | 1 (2.9) |
| Low-moderate dose steroid, n (%) | 1 (2.9) |
| Bendamustine, n (%) | 3 (8.6) |
| Fludarabine, n (%) | 1 (2.9) |
| Cladribine, n (%) | 0 (0.0) |
| Anthracycline-based Chemotherapy, n (%) | 2 (5.7) |
| Rituximab, n (%) | 6 (17.1) |
| Obinutuzumab, n (%) | 7 (20.0) |
| Ibritumomab tiuxetan, n (%) | 0 (0.0) |
| Lenalidomide, n (%) | 1 (2.9) |
| Radiation / Operation, n (%) | 1 (2.9) / 0 (0.0) |
| Eradication Tx for Helicobacter. / Other, n (%) | 0 (0.0) / 2 (5.7) |

**(15) ITL n=4**

| **Variable** | **Patients (n=4)** |
| --- | --- |
| **Treatments for hematological disease within the last year** | |
| No treatment, observation, n (%) | 0 (0.0) |
| High dose steroid, n (%) | 1 (25.0) |
| Low-moderate dose steroid, n (%) | 1 (25.0) |
| Anthracycline-based Chemotherapy, n (%) | 2 (50.0) |
| Interferon γ-1a, n (%) | 0 (0.0) |
| Alemtuzumab, n (%) | 0 (0.0) |
| Radiation, n (%) | 1 (25.0) |
| Others, n (%) | 3 (75.0) |
| **Ongoing treatments for hematological disease at COVID-19 Dx** | |
| No treatment, observation, n (%) | 2 (50.0) |
| High dose steroid, n (%) | 0 (0.0) |
| Low-moderate dose steroid, n (%) | 0 (0.0) |
| Anthracycline-based Chemotherapy, n (%) | 0 (0.0) |
| Interferon γ-1a, n (%) | 0 (0.0) |
| Alemtuzumab, n (%) | 0 (0.0) |
| Radiation, n (%) | 0 (0.0) |
| Others, n (%) | 2 (50.0) |

**(16) ITP n=20**

| **Variable** | **Patients (n=20)** |
| --- | --- |
| **Treatments for hematological disease within the last year** | |
| No treatment, observation, n (%) | 6 (30.0) |
| Transfusion PC, n (%) | 4 (20.0) |
| High dose steroid, n (%) | 3 (15.0) |
| Low-moderate steroid, n (%) | 12 (60.0) |
| Intravenous immunoglobulin, n (%) | 4 (20.0) |
| Rituximab, n (%) | 3 (15.0) |
| Eltrombopag, n (%) | 8 (40.0) |
| Romiplostim, n (%) | 2 (10.0) |
| Splenectomy, n (%) | 1 (5.0) |
| Others, n (%) | 5 (14.0) |
| **Ongoing treatments for hematological disease at COVID-19 Dx** | |
| No treatment, observation, n (%) | 1 (5.0) |
| Transfusion PC, n (%) | 2 (10.0) |
| High dose steroid, n (%) | 0 (0.0) |
| Low-moderate steroid, n (%) | 8 (40.0) |
| Intravenous immunoglobulin, n (%) | 1 (5.0) |
| Rituximab, n (%) | 1 (5.0) |
| Eltrombopag, n (%) | 5 (25.0) |
| Romiplostim, n (%) | 1 (5.0) |
| Splenectomy, n (%) | 1 (5.0) |
| Others, n (%) | 1 (5.0) |

**(17) LPL n=7**

| **Variable** | **Patients (n=7)** |
| --- | --- |
| **Treatments for hematological disease within the last year** | |
| No treatment, observation, n (%) | 0 (0.0) |
| High dose steroid, n (%) | 2 (28.6) |
| Low-moderate dose steroid, n (%) | 1 (14.3) |
| Bendamustine, n (%) | 1 (14.3) |
| Fludarabine, n (%) | 0 (0.0) |
| Cyclophosphamide, n (%) | 0 (0.0) |
| Rituximab, n (%) | 1 (14.3) |
| Bortezomib, n (%) | 1 (14.3) |
| Tirabrutinib, n (%) | 4 (57.1) |
| Radiation, n (%) | 0 (0.0) |
| Plasma exchange, n (%) | 0 (0.0) |
| Others, n (%) | 0 (0.0) |
| **Ongoing treatments for hematological disease at COVID-19 Dx** | |
| No treatment, observation, n (%) | 3 (42.9) |
| High dose steroid, n (%) | 0 (0.0) |
| Low-moderate dose steroid, n (%) | 1 (14.3) |
| Bendamustine, n (%) | 0 (0.0) |
| Fludarabine, n (%) | 0 (0.0) |
| Cyclophosphamide, n (%) | 0 (0.0) |
| Rituximab, n (%) | 0 (0.0) |
| Bortezomib, n (%) | 0 (0.0) |
| Tirabrutinib, n (%) | 3 (42.9) |
| Radiation, n (%) | 0 (0.0) |
| Plasma exchange, n (%) | 0 (0.0) |
| Others, n (%) | 0 (0.0) |

**(18) MCL n=5**

| **Variable** | **Patients (n=5)** |
| --- | --- |
| **Treatments for hematological disease within the last year** | |
| No treatment, observation, n (%) | 1 (20.0) |
| High dose steroid, n (%) | 1 (20.0) |
| Low-moderate dose steroid, n (%) | 0 (0.0) |
| Anthracycline-based Chemotherapy, n (%) | 1 (20.0) |
| Bendamustine, n (%) | 3 (60.0) |
| Dose dense anthracycline based Tx, n (%) | 2 (40.0) |
| High dose AraC-based therapy, n (%) | 1 (20.0) |
| Fludarabine / Cladribine, n (%) | 0 (0.0) / 0 (0.0) |
| Platinum based chemotherapy, n (%) | 0 (0.0) |
| Rituximab, n (%) | 3 (60.0) |
| Bortezomib based regimen, n (%) | 0 (0.0) |
| Ibrutinib, n (%) | 1 (20.0) |
| Radiation, n (%) | 0 (0.0) |
| Others, n (%) | 0 (0.0) |
| **Ongoing treatments for hematological disease at COVID-19 Dx** | |
| No treatment, observation, n (%) | 1 (20.0) |
| High dose steroid, n (%) | 0 (0.0) |
| Low-moderate dose steroid, n (%) | 0 (0.0) |
| Anthracycline-based Chemotherapy, n (%) | 0 (0.0) |
| Bendamustine, n (%) | 3 (60.0) |
| Dose dense anthracycline based Tx, n (%) | 0 (0.0) |
| High dose AraC-based therapy, n (%) | 0 (0.0) |
| Fludarabine / Cladribine, n (%) | 0 (0.0) / 0 (0.0) |
| Platinum based chemotherapy, n (%) | 0 (0.0) |
| Rituximab, n (%) | 2 (40.0) |
| Bortezomib based regimen, n (%) | 0 (0.0) |
| Ibrutinib, n (%) | 0 (0.0) |
| Radiation, n (%) | 0 (0.0) |
| Others, n (%) | 0 (0.0) |

**(19) MDS n=36**

| **Variable** | **Patients (n=36)** |
| --- | --- |
| **Treatments for hematological disease within the last year** | |
| No treatment, observation, n (%) | 5 (13.9) |
| Transfusion RBC / PC, n (%) | 25 (69.4) / 14 (38.9) |
| G-CSF, n (%) | 1 (2.8) |
| Iron chelation, n (%) | 5 (13.9) |
| ESA, n (%) | 1 (2.8) |
| Danazol / Metenolone, n (%) | 1 (2.8) / 1 (2.8) |
| Azacitidine, n (%) | 18 (50.0) |
| Lenalidomide, n (%) | 0 (0.0) |
| Others, n (%) | 4 (11.1) |
| **Ongoing treatments for hematological disease at COVID-19 Dx** | |
| No treatment, observation, n (%) | 6 (16.7) |
| Transfusion RBC / PC, n (%) | 19 (52.8) / 10 (27.8) |
| G-CSF, n (%) | 1 (2.8) |
| Iron chelation, n (%) | 4 (11.1) |
| ESA, n (%) | 1 (2.8) |
| Danazol / Metenolone, n (%) | 1 (2.8) / 1(2.8) |
| Azacitidine, n (%) | 14 (38.9) |
| Lenalidomide, n (%) | 0 (0.0) |
| Others, n (%) | 5 (13.9) |

**(20) MF n=4**

| **Variable** | **Patients (n=4)** |
| --- | --- |
| **Treatments for hematological disease within the last year** | |
| No treatment, observation, n (%) | 1 (25.0) |
| Transfusion RBC / PC, n (%) | 1 (25.0) / 0 (0.0) |
| Iron chelation, n (%) | 1 (25.0) |
| Hydroxyurea, n (%) | 0 (0.0) |
| Danazol / Metenolone, n (%) | 0 (0.0) / 0 (0.0) |
| Ruxolitinib, n (%) | 1 (25.0) |
| Others, n (%) | 1 (25.0) |
| **Ongoing treatments for hematological disease at COVID-19 Dx** | |
| No treatment, observation, n (%) | 1 (25.0) |
| Transfusion RBC / PC, n (%) | 1 (25.0) / 0 (0.0) |
| Iron chelation, n (%) | 0 (0.0) |
| Hydroxyurea, n (%) | 0 (0.0) |
| Danazol / Metenolone, n (%) | 0 (0.0) / 0 (0.0) |
| Ruxolitinib, n (%) | 2 (50.0) |
| Others, n (%) | 1 (25.0) |

**(21) MM n=46**

| **Variable** | **Patients (n=46)** |
| --- | --- |
| **Treatments for hematological disease within the last year** | |
| No treatment, observation, n (%) | 7 (15.2) |
| DEX / PSL, n (%) | 37 (80.4) / 5 (10.9) |
| Cyclophosphamide, n (%) | 6 (13.0) |
| Vincristine, n (%) | 2 (4.4) |
| Doxorubicin, n (%) | 2 (4.4) |
| Melphalan, n (%) | 3 (6.5) |
| Daratumumab / Isatuximab, n (%) | 14 (30.4) / 3 (6.5) |
| Elotuzumab, n (%) | 6 (13.0) |
| Thalidomide / Lenalidomide / Pomalidomide, n (%) | 1(2.2)/29(63.0)/11(23.9) |
| Bortezomib / Carfilzomib / Ixazomib, n (%) | 14(30.4)/11(23.9)/6(13.0) |
| Panobinostat, n (%) | 0 (0.0) |
| Others, n (%) | 2 (4.4) |
| **Ongoing treatments for hematological disease at COVID-19 Dx** | |
| No treatment, observation, n (%) | 10 (21.7) |
| DEX / PSL, n (%) | 32 (69.6) / 2 (4.3) |
| Cyclophosphamide, n (%) | 4 (8.7) |
| Vincristine, n (%) | 1 (2.2) |
| Doxorubicin, n (%) | 1 (2.2) |
| Melphalan, n (%) | 0 (0.0) |
| Daratumumab / Isatuximab, n (%) | 10 (21.7) / 2 (4.3) |
| Elotuzumab, n (%) | 3 (6.5) |
| Thalidomide / Lenalidomide / Pomalidomide, n (%) | 0(0.0)/18(39.1)/7(15.2) |
| Bortezomib / Carfilzomib / Ixazomib, n (%) | 9(19.6)/5(10.9)/1(2.2) |
| Panobinostat, n (%) | 0 (0.0) |
| Others, n (%) | 0 (0.0) |

**(22) MPAL n=1**

| **Variable** | **Patients (n=1)** |
| --- | --- |
| **Treatments for hematological disease within the last year** | |
| No treatment, observation, n (%) | 0 (0.0) |
| Intensive induction therapy, n (%) | 1 (100.0) |
| Consolidation therapy, n (%) | 1 (100.0) |
| Maintenance therapy, n (%) | 0 (0.0) |
| HyperCVAD/MA, n (%) | 1 (100.0) |
| Low dose palliative therapy, n (%) | 0 (0.0) |
| Nelarabine, n (%) | 0 (0.0) |
| Blinatumomab, n (%) | 0 (0.0) |
| Inotuzumab ozogamicin, n (%) | 0 (0.0) |
| CD19-CART, n (%) | 0 (0.0) |
| Others, n (%) | 1 (100.0) |
| **Ongoing treatments for hematological disease at COVID-19 Dx** | |
| No treatment, observation, n (%) | 1 (100.0) |
| Intensive induction therapy, n (%) | 0 (0.0) |
| Consolidation therapy, n (%) | 0 (0.0) |
| Maintenance therapy, n (%) | 0 (0.0) |
| HyperCVAD/MA, n (%) | 0 (0.0) |
| Low dose palliative therapy, n (%) | 0 (0.0) |
| Nelarabine, n (%) | 0 (0.0) |
| Blinatumomab, n (%) | 0 (0.0) |
| Inotuzumab ozogamicin, n (%) | 0 (0.0) |
| CD19-CART, n (%) | 0 (0.0) |
| Others, n (%) | 0 (0.0) |

**(23) MPN (PV,ET) n=12**

| **Variable** | **Patients (n=12)** |
| --- | --- |
| **Treatments for hematological disease within the last year** | |
| No treatment, observation, n (%) | 2 (16.7) |
| Phlebotomy, n (%) | 0 (0.0) |
| Hydroxyurea, n (%) | 7 (58.3) |
| Anagrelide, n (%) | 1 (8.3) |
| Ruxolitinib, n (%) | 1 (8.3) |
| Others, n (%) | 3 (25.0) |
| **Ongoing treatments for hematological disease at COVID-19 Dx** | |
| No treatment, observation, n (%) | 2 (16.7) |
| Phlebotomy, n (%) | 0 (0.0) |
| Hydroxyurea, n (%) | 7 (58.3) |
| Anagrelide, n (%) | 1 (8.3) |
| Ruxolitinib, n (%) | 1 (8.3) |
| Others, n (%) | 2 (16.7) |

**(24) PCNSL n=7**

| **Variable** | **Patients (n=7)** |
| --- | --- |
| **Treatments for hematological disease within the last year** | |
| No treatment, observation, n (%) | 1 (14.3) |
| High dose steroid, n (%) | 5 (71.4) |
| Low-moderate dose steroid, n (%) | 1 (14.3) |
| High dose MTX based regimen, n (%) | 6 (85.7) |
| High dose AraC based regimen, n (%) | 6 (85.7) |
| Rituximab, n (%) | 5 (71.4) |
| Tirabrutinib, n (%) | 3 (42.9) |
| Radiation, n (%) | 3 (42.9) |
| Intrathecal chemotherapy, n (%) | 0 (0.0) |
| Others, n (%) | 0 (0.0) |
| **Ongoing treatments for hematological disease at COVID-19 Dx** | |
| No treatment, observation, n (%) | 3 (42.9) |
| High dose steroid, n (%) | 2 (28.6) |
| Low-moderate dose steroid, n (%) | 0 (0.0) |
| High dose MTX based regimen, n (%) | 2 (28.6) |
| High dose AraC based regimen, n (%) | 0 (0.0) |
| Rituximab, n (%) | 1 (14.3) |
| Tirabrutinib, n (%) | 2 (28.6) |
| Radiation, n (%) | 0 (0.0) |
| Intrathecal chemotherapy, n (%) | 0 (0.0) |
| Others, n (%) | 0 (0.0) |

**(25) Ph(ｰ)ALL n=5**

| **Variable** | **Patients (n=5)** |
| --- | --- |
| **Treatments for hematological disease within the last year** | |
| No treatment, observation, n (%) | 1 (20.0) |
| Intensive induction therapy, n (%) | 2 (40.0) |
| Consolidation therapy, n (%) | 2 (40.0) |
| Maintenance therapy, n (%) | 3 (60.0) |
| HyperCVAD/MA, n (%) | 0 (0.0) |
| Low dose palliative therapy, n (%) | 0 (0.0) |
| Blinatumomab, n (%) | 1 (20.0) |
| Inotuzumab ozogamicin, n (%) | 0 (0.0) |
| CD19-CART, n (%) | 0 (0.0) |
| Others, n (%) | 0 (0.0) |
| **Ongoing treatments for hematological disease at COVID-19 Dx** | |
| No treatment, observation, n (%) | 3 (60.0) |
| Intensive induction therapy, n (%) | 1 (20.0) |
| Consolidation therapy, n (%) | 1 (20.0) |
| Maintenance therapy, n (%) | 0 (0.0) |
| HyperCVAD/MA, n (%) | 0 (0.0) |
| Low dose palliative therapy, n (%) | 0 (0.0) |
| Blinatumomab, n (%) | 0 (0.0) |
| Inotuzumab ozogamicin, n (%) | 0 (0.0) |
| CD19-CART, n (%) | 0 (0.0) |
| Others, n (%) | 0 (0.0) |

**(26) Ph(+)ALL n=6**

| **Variable** | **Patients (n=6)** |
| --- | --- |
| **Treatments for hematological disease within the last year** | |
| No treatment, observation, n (%) | 1 (16.7) |
| Intensive induction therapy, n (%) | 3 (50.0) |
| Consolidation therapy, n (%) | 3 (50.0) |
| Maintenance therapy, n (%) | 2 (33.3) |
| HyperCVAD/MA, n (%) | 2 (33.3) |
| Low dose palliative therapy, n (%) | 0 (0.0) |
| Blinatumomab, n (%) | 0 (0.0) |
| Inotuzumab ozogamicin, n (%) | 0 (0.0) |
| Imatinib / Dasatinib / Ponatinib, n (%) | 2(33.3) / 1(16.7) / 3(50.0) |
| CD19-CART, n (%) | 0 (0.0) |
| Others, n (%) | 0 (0.0) |
| **Ongoing treatments for hematological disease at COVID-19 Dx** | |
| No treatment, observation, n (%) | 3 (50.0) |
| Intensive induction therapy, n (%) | 0 (0.0) |
| Consolidation therapy, n (%) | 1 (16.7) |
| Maintenance therapy, n (%) | 2 (33.3) |
| HyperCVAD/MA, n (%) | 0 (0.0) |
| Low dose palliative therapy, n (%) | 0 (0.0) |
| Blinatumomab, n (%) | 0 (0.0) |
| Inotuzumab ozogamicin, n (%) | 0 (0.0) |
| Imatinib / Dasatinib / Ponatinib, n (%) | 1(16.7) / 0(0.0) / 1(16.7) |
| CD19-CART, n (%) | 0 (0.0) |
| Others, n (%) | 0 (0.0) |

**(27) PNH n=3**

| **Variable** | **Patients (n=3)** |
| --- | --- |
| **Treatments for hematological disease within the last year** | |
| No treatment, observation, n (%) | 0 (0.0) |
| Transfusion RBC / PC, n (%) | 0 (0.0) / 0 (0.0) |
| High dose steroid, n (%) | 0 (0.0) |
| Low-moderate steroid, n (%) | 0 (0.0) |
| Metenolone, n (%) | 1 (33.3) |
| Eclizumab, n (%) | 1 (33.3) |
| Ravulizumab, n (%) | 1 (33.3) |
| Others, n (%) | 1 (33.3) |
| **Ongoing treatments for hematological disease at COVID-19 Dx** | |
| No treatment, observation, n (%) | 0 (0.0) |
| Transfusion RBC / PC, n (%) | 0 (0.0) |
| High dose steroid, n (%) | 0 (0.0) |
| Low-moderate steroid, n (%) | 0 (0.0) |
| Metenolone, n (%) | 1 (33.3) |
| Eclizumab, n (%) | 1 (33.3) |
| Ravulizumab, n (%) | 1 (33.3) |
| Others, n (%) | 1 (33.3) |

**(28) POEMS n=3**

| **Variable** | **Patients (n=3)** |
| --- | --- |
| **Treatments for hematological disease within the last year** | |
| No treatment, observation, n (%) | 0 (0.0) |
| DEX / PSL, n (%) | 1 (33.3) / 1 (33.3) |
| Cyclophosphamide, n (%) | 0 (0.0) |
| Vincristine, n (%) | 0 (0.0) |
| Doxorubicin, n (%) | 0 (0.0) |
| Melphalan, n (%) | 0 (0.0) |
| Daratumumab / Isatuximab, n (%) | 0 (0.0) / 0 (0.0) |
| Elotuzumab, n (%) | 0 (0.0) |
| Thalidomide / Lenalidomide / Pomalidomide, n (%) | 2(66.7)/2(66.7)/0(0.0) |
| Bortezomib / Carfilzomib / Ixazomib, n (%) | 1(33.3)/0(0.0)/1(33.3) |
| Panobinostat, n (%) | 0 (0.0) |
| Others, n (%) | 0 (0.0) |
| **Ongoing treatments for hematological disease at COVID-19 Dx** | |
| No treatment, observation, n (%) | 0 (0.0) |
| DEX / PSL, n (%) | 1 (33.3) / 1 (33.3) |
| Cyclophosphamide, n (%) | 0 (0.0) |
| Vincristine, n (%) | 0 (0.0) |
| Doxorubicin, n (%) | 0 (0.0) |
| Melphalan, n (%) | 0 (0.0) |
| Daratumumab / Isatuximab, n (%) | 0 (0.0) / 0 (0.0) |
| Elotuzumab, n (%) | 0 (0.0) |
| Thalidomide / Lenalidomide / Pomalidomide, n (%) | 1(33.3)/1(33.3)/0(0.0) |
| Bortezomib / Carfilzomib / Ixazomib, n (%) | 1(33.3)/0(0.0)/1(33.3) |
| Panobinostat, n (%) | 0 (0.0) |
| Others, n (%) | 0 (0.0) |

**(29) PRCA n=4**

| **Variable** | **Patients (n=4)** |
| --- | --- |
| **Treatments for hematological disease within the last year** | |
| No treatment, observation, n (%) | 0 (0.0) |
| Transfusion RBC , n (%) | 1 (25.0) |
| High dose steroid, n (%) | 0 (0.0) |
| Low-moderate steroid, n (%) | 1 (25.0) |
| CyA, n (%) | 3 (75.0) |
| Cyclophosphamide, n (%) | 0 (0.0) |
| Others, n (%) | 0 (0.0) |
| **Ongoing treatments for hematological disease at COVID-19 Dx** | |
| No treatment, observation, n (%) | 0 (0.0) |
| Transfusion RBC , n (%) | 1 (25.0) |
| High dose steroid, n (%) | 0 (0.0) |
| Low-moderate steroid, n (%) | 0 (0.0) |
| CyA, n (%) | 3 (75.0) |
| Cyclophosphamide, n (%) | 0 (0.0) |
| Others, n (%) | 0 (0.0) |

**(30) T-ALL n=1**

| **Variable** | **Patients (n=1)** |
| --- | --- |
| **Treatments for hematological disease within the last year** | |
| No treatment, observation, n (%) | 0 (0.0) |
| Intensive induction therapy, n (%) | 0 (0.0) |
| Consolidation therapy, n (%) | 1 (100.0) |
| Maintenance therapy, n (%) | 0 (0.0) |
| HyperCVAD/MA, n (%) | 0 (0.0) |
| Low dose palliative therapy, n (%) | 0 (0.0) |
| Nelarabine, n (%) | 0 (0.0) |
| Imatinib / Dasatinib / Ponatinib, n (%) | 0(0.0) / 0(0.0) / 0(0.0) |
| Others, n (%) | 0 (0.0) |
| **Ongoing treatments for hematological disease at COVID-19 Dx** | |
| No treatment, observation, n (%) | 1 (0.0) |
| Intensive induction therapy, n (%) | 0 (0.0) |
| Consolidation therapy, n (%) | 0 (0.0) |
| Maintenance therapy, n (%) | 0 (0.0) |
| HyperCVAD/MA, n (%) | 0 (0.0) |
| Low dose palliative therapy, n (%) | 0 (0.0) |
| Nelarabine, n (%) | 0 (0.0) |
| Imatinib / Dasatinib / Ponatinib, n (%) | 0(0.0) / 0(0.0) / 0(0.0) |
| Others, n (%) | 0 (0.0) |

**(31) Thalassemia n=1**

| **Variable** | **Patients (n=1)** |
| --- | --- |
| **Treatments for hematological disease within the last year** | |
| No treatment, observation, n (%) | 0 (0.0) |
| Transfusion RBC , n (%) | 1 (100) |
| Deferoxamine, n (%) | 0 (0.0) |
| Deferasirox, n (%) | 0 (0.0) |
| Others, n (%) | 0 (0.0) |
| **Ongoing treatments for hematological disease at COVID-19 Dx** | |
| No treatment, observation, n (%) | 1 (0.0) |
| Transfusion RBC , n (%) | 0 (0.0) |
| Deferoxamine, n (%) | 0 (0.0) |
| Deferasirox, n (%) | 0 (0.0) |
| Others, n (%) | 0 (0.0) |

**(32) TTP n=1**

| **Variable** | **Patients (n=1)** |
| --- | --- |
| **Treatments for hematological disease within the last year** | |
| No treatment, observation, n (%) | 0 (0.0) |
| Transfusion FFP, n (%) | 0 (0.0) |
| High dose steroid, n (%) | 0 (0.0) |
| Low-moderate steroid, n (%) | 1 (100) |
| Plasma exchange, n (%) | 0 (0.0) |
| Rituximab, n (%) | 0 (0.0) |
| Others, n (%) | 0 (0.0) |
| **Ongoing treatments for hematological disease at COVID-19 Dx** | |
| No treatment, observation, n (%) | 1 (100) |
| Transfusion FFP, n (%) | 0 (0.0) |
| High dose steroid, n (%) | 0 (0.0) |
| Low-moderate steroid, n (%) | 0 (0.0) |
| Plasma exchange, n (%) | 0 (0.0) |
| Rituximab, n (%) | 0 (0.0) |
| Others, n (%) | 0 (0.0) |

**(33) wAIHA n=4**

| **Variable** | **Patients (n=4)** |
| --- | --- |
| **Treatments for hematological disease within the last year** | |
| No treatment, observation, n (%) | 0 (0.0) |
| Transfusion RBC, n (%) | 0 (0.0) |
| High dose steroid, n (%) | 0 (0.0) |
| Low-moderate steroid, n (%) | 4 (100) |
| Cyclophosphamide, n (%) | 0 (0.0) |
| Azathioprine, n (%) | 0 (0.0) |
| Rituximab, n (%) | 0 (0.0) |
| Splenectomy, n (%) | 0 (0.0) |
| Others, n (%) | 1 (25.0) |
| **Ongoing treatments for hematological disease at COVID-19 Dx** | |
| No treatment, observation, n (%) | 1 (25.0) |
| Transfusion RBC, n (%) | 0 (0.0) |
| High dose steroid, n (%) | 0 (0.0) |
| Low-moderate steroid, n (%) | 3 (75.0) |
| Cyclophosphamide, n (%) | 0 (0.0) |
| Azathioprine, n (%) | 0 (0.0) |
| Rituximab, n (%) | 0 (0.0) |
| Splenectomy, n (%) | 0 (0.0) |
| Others, n (%) | 1 (25.0) |

**Table S2 (1)–(33)** Ongoing treatments for hematological disease or treatments given within the 1 year before COVID-19 diagnosis. **1** AA, aplastic anemia; **2** AML, acute myeloid leukemia; **3** APL, acute promyelocytic leukemia; **4** ATL(L), adult T-cell leukemia-(lymphoma); **5** AUL, acute undifferentiated leukemia; **6** CAD, cold agglutinin disease; **7** CLL, chronic lymphocytic leukemia; **8** CML, chronic myeloid leukemia; **9** CMML, chronic myelomonocytic leukemia; **10** ENTL, extranodal NK/T cell lymphoma; **11** aggressive BL, aggressive B-cell lymphoma; **12** aggressive TL, aggressive T-cell lymphoma; **13** HL, Hodgkin lymphoma; **14** IBL, indolent B-cell lymphoma; **15** ITL, indolent T-cell lymphoma; **16** ITP, idiopathic thrombocytopenic purpura; **17** LPL, lymphoplasmacytic lymphoma; **18** MCL, mantle cell lymphoma; **19** MDS, myelodysplastic syndrome; **20** MF, myelofibrosis; **21** MM, multiple myeloma; **22** MPAL, mixed-phenotype acute leukemia; **23** MPN, myeloproliferative neoplasm; **24** PCNSL, primary central nervous system lymphoma; **25** Philadelphia (Ph)-negative ALL (acute lymphocytic leukemia); **26** Ph-positive ALL; **27** PNH, paroxysmal nocturnal hemoglobinuria; **28** POEMS syndrome; **29** PRCA, pure red cell aplasia; **30** T-ALL, T-cell lymphoblastic leukemia; **31** thalassemia; **32** TTP, thrombotic thrombocytopenic purpura; **33** wAIHA, warm autoimmune hemolytic anemia

**Table S3 Treatments and supportive care for COVID-19**

| Treatments for COVID-19 | | Supportive care for COVID-19 | |
| --- | --- | --- | --- |
| No treatment for COVID-19, n (%) | 80 (21.8%) | No supportive oxygen required, n (%) | 210 (57.2%) |
| Dexamethasone (including alternative glucocorticoids), n (%) | 137 (37.3%) | 1–5 L/min supportive oxygen required, n (%) | 146 (39.8%) |
| Ciclesonide, n (%) | 7 (1.9%) | >5 L/min (including nasal high flow), n (%) | 79 (21.5%) |
| Losartan, n (%) | 1 (0.3%) | Days from diagnosis to need for oxygen, median (range), days | 2 (-349–45) |
| Azithromycin, n (%) | 5 (1.4%) | Duration of oxygen, median (range), days | 11 (0–229) |
| Ivermectin, n (%) | 3 (0.8%) | Non-invasive mechanical ventilation (NPPV etc. ), n (%) | 11 (3.0%) |
| Famotidine, n (%) | 6 (1.6%) | Invasive mechanical ventilation, n (%) | 30 (8.2%) |
| Ribavirin, n (%) | 0 (0.0%) | Days from oxygen to ventilator start, median (range), days | 4 (0–12) |
| Remdesivir, n (%) | 148 (40.3%) | Duration of ventilation, median (range), days | 20 (5–69) |
| Lopinavir + ritonavir, n (%) | 1 (0.3%) | Vasopressors and/or inotropes, n (%) | 18 (4.9%) |
| Favipiravir, n (%) | 57 (15.5%) | Renal replacement therapy, n (%) | 8 (2.2%) |
| Nelfinavir, n (%) | 0 (0.0%) | ECMO, n (%) | 3 (0.8%) |
| Hydroxychroloquine, n (%) | 2 (0.5%) | Days from diagnosis to ECMO start, median (range), days | 7 (0–19) |
| Bamlanivimab, n (%) | 0 (0.0%) | Duration of ECMO, median (range), days | 10 (9–22) |
| Tocilizumab, n (%) | 23 (6.3%) | Anticoagulant therapy, n (%) | 89 (24.3%) |
| Sarilumab, n (%) | 0 (0.0%) | LMWH, n (%) | 24 (6.5%) |
| Siltuximab, n (%) | 0 (0.0%) | UFH, n (%) | 53 (14.4%) |
| Baricitinib, n (%) | 14 (3.8%) | Antithrombin, n (%) | 3 (0.8%) |
| Nafamostat mesylate, n (%) | 11 (3.0%) | rTM, n (%) | 4 (1.1%) |
| Camostat mesylate, n (%) | 1 (0.3%) | DOAC |  |
| Adrenomedullin, n (%) | 0 (0.0%) | - Fondaparinux, n (%) | 0 (0.0%) |
| Intravenous immunoglobulin, n (%) | 18 (4.9%) | - Apixaban, n (%) | 7 (1.9%) |
| Mesenchymal stem cells, n (%) | 0 (0.0%) | - Rivaroxaban, n (%) | 1 (0.3%) |
| Convalescent plasma, n (%) | 0 (0.0%) | - Edoxaban, n (%) | 11 (3.0%) |
| Unknown/other, n (%) | 7 (1.9%)/111 (30.3%) | - Dabigatran, n (%) | 0 (0.0%) |
|  |  | Warfarin, n (%) | 2 (0.5%) |
|  |  | SPIs, n (%) | 3 (0.8%) |
|  |  | Heparinoid, n (%) | 0 (0.0%) |
|  |  | Transfusion, n (%) | 57 (15.5%) |
|  |  | Others/unknown, n (%) | 16 (4.4%)/6 (1.6%) |

NPPV, noninvasive positive pressure ventilation; ECMO, extracorporeal membrane oxygenation; LMWH, low-molecular-weight heparin; UFH, unfractionated heparin; rTM, recombinant thrombomodulin; DOAC, direct oral anticoagulant; SPIs, synthetic protease inhibitors.

**Table S4 Univariate and multivariate analyses of 60-day OS (in a cohort excluding benign disease)**

| Risk factor | Univariate analysis | | Multivariate analysis | |
| --- | --- | --- | --- | --- |
|  | 60-day OS [%], 95%CI | P-value | HR (95%CI) | P-value |
| Age (>60) | 81.7 (75.9–86.2) | 0.012 |  | 0.870 |
| (≤60) | 92.3 (85.2–96.1) |  | Reference |  |
| Alb (≤3.3 g/dl) | 64.0 (52.9–73.1) | <0.001 | **4.165 (2.002–8.666)** | **<0.001** |
| (>3.3 g/dl) | 91.5 (86.0–94.9) |  | Reference |  |
| ASH classification (severe) | 63.2 (37.9–80.4) | 0.004 |  | 0.997 |
| (mild and moderate) | 86.5 (82.1–89.9) |  | Reference |  |
| MHLW classification (severe) | 58.8 (32.5–77.8) | 0.001 |  | 0.997 |
| (mild and moderate I, II) | 86.6 (82.2–90.0) |  | Reference |  |
| BMI (>30 kg/m^2^) | 75.0 (31.5–93.1) | 0.470 |  |  |
| (≤30 kg/m^2^) | 85.3 (80.9–88.8) |  |  |  |
| Current or former smoking | 86.8 (79.0–91.8) | 0.530 |  |  |
| Never | 84.2 (78.6–88.5) |  |  |  |
| MDS | 80.6 (63.5–90.2) | 0.405 |  |  |
| No MDS | 85.7 (81.1–89.3) |  |  |  |
| Leukemia | 89.1 (75.8–95.3) | 0.397 |  |  |
| No leukemia | 84.5 (79.6–88.2) |  |  |  |
| Lymphoma | 83.5 (76.0–88.8) | 0.496 |  |  |
| No lymphoma | 86.3 (80.5–90.5) |  |  |  |
| PCD | 81.4 (68.9–89.2) | 0.369 |  |  |
| No PCD | 86.0 (81.2–89.6) |  |  |  |
| PS (>2) | 66.8 (55.2–76.1) | <0.001 |  | 0.384 |
| (≤2) | 91.0 (86.7–94.0) |  | Reference |  |
| Relapsed or refractory | 66.1 (52.1–76.8) | <0.001 |  | 0.093 |
| Except for the above | 89.1 (84.7–92.3) |  | Reference |  |
| Ongoing therapy at diagnosis | 83.4 (77.1–88.1) | 0.318 |  |  |
| Except for the above | 87.3 (80.7–91.8) |  |  |  |
| Oxygen required | 68.0 (59.6–75.0) | <0.001 | **14.72 (3.421–63.33)** | **<0.001** |
| Except for the above | 98.4 (95.0–99.5) |  | Reference |  |
| Neutrophils (<1000/µL) | 91.7 (76.3–97.2) | 0.124 |  | 0.114 |
| (≥1000/µL) | 81.2 (75.4–85.7) |  | Reference |  |
| Lymphocytes (<1000/µL) | 80.5 (73.9–85.6) | 0.186 |  | 0.781 |
| (≥1000/µL) | 87.6 (78.2–93.1) |  | Reference |  |
| CRP (>7.0 mg/dl) | 62.7 (49.4–73.4) | <0.001 |  | 0.297 |
| (≤7.0 mg/dl) | 88.3 (82.9–92.1) |  | Reference |  |

OS, overall survival; ASH, American Society of Hematology; MHLW, Ministry of Health, Labour and Welfare in Japan; BMI, body mass Index; MDS, myelodysplastic syndromes; PCD, plasma cell dyscrasia; PS, performance status; CRP, C-reactive protein.
